# PLAA suppresses ovarian cancer metastasis via METTL3-mediated m^6^A modification of TRPC3 mRNA

**DOI:** 10.1038/s41388-022-02411-w

**Published:** 2022-07-22

**Authors:** Zhangjin Shen, Lingkai Gu, Yuwan Liu, Lingfang Wang, Jiawei Zhu, Sangsang Tang, Xinyi Wei, Jiaying Wang, Songfa Zhang, Xinyu Wang, Xiaodong Cheng, Xing Xie, Weiguo Lu

**Affiliations:** 1 Women’s Reproductive Health Laboratory of Zhejiang Province, Women’s Hospital, Zhejiang University School of Medicine, Hangzhou, 310006 Zhejiang China; 2 Department of Gynecologic Oncology, Women’s Hospital, Zhejiang University School of Medicine, Hangzhou, 310006 Zhejiang China; 3 Department of Obstetrics and Gynecology, the Seventh Medical Centre, Chinese PLA General Hospital, Beijing, 100853 China; 4 Cancer Center, Zhejiang University, Hangzhou, 310058 Zhejiang China; 5 Department of Obstetrics and Gynecology, The First Affiliated Hospital, Zhejiang University School of Medicine, Hangzhou, 310003 Zhejiang China; 6 Zhejiang Provincial Key Laboratory of Precision Diagnosis and Therapy for Major Gynecological Diseases, Women’s Hospital, Zhejiang University School of Medicine, Hangzhou, 310006 Zhejiang China

**Keywords:** Ovarian cancer, Cell migration, Epigenetics

## Abstract

Wide metastasis contributes to a high death rate in ovarian cancer, and understanding of the molecular mechanism helps to find effective targets for metastatic ovarian cancer therapy. It has been found that phospholipase A2-activating protein (PLAA) is inactivated in some cancers, but its role in cancer metastasis remains unknown. Here, we found that PLAA was significantly downregulated in ovarian cancer highly metastatic cell lines and patients, and the low expression of PLAA was associated with poorer prognosis and high-risk clinicopathological features of patients. PLAA inhibited the migration and invasion of ovarian cancer cells and metastasis of transplanted tumor in the orthotopic xenograft mouse model. Meanwhile, PLAA inhibited metastasis of ovarian cancer by inhibiting transient receptor potential channel canonical 3 (TRPC3)-mediated the intracellular Ca^2+^ level. Mechanistically, PLAA inhibited methyltransferase-like 3 (METTL3) expression through the ubiquitin-mediated degradation, and METTL3 stabilized TRPC3 mRNA expression via N6-methyladenosine (m^6^A) modification. Our study verified the function and mechanism of the PLAA-METTL3-TRPC3 axis involved in ovarian cancer metastasis, with a view to providing a potential therapeutic approach for ovarian cancer.

## Introduction

Epithelial ovarian cancer is the most lethal gynecological malignancy worldwide [1, 2]. One of the most important biological characteristics of ovarian cancer is its insidious and rapid spread from the primary tumor into the peritoneal cavity, involving almost all abdominal organs, and distant organs, which contributes to the high death rate of disease [3, 4]. Accordingly, it is urgent to understand the biological process of ovarian cancer metastasis, so as to find effective therapeutic target for metastatic ovarian cancer.

Multiple signal pathways are involved in cancer metastasis. We thus profiled a pair of epithelial ovarian cancer cell lines with different metastatic potentials [5] by using proteomics, and discovered that phospholipase A2-activating protein (PLAA) was downregulated in highly metastatic ovarian cancer cells. PLAA is encoded by gene PLAA that is located on chromosome 9p21 [6]. It has been verified that PLAA has a highly homologous sequence with yeast Ufd3/Doa1 [7], but little is known about its biological function so far, such as the endo-lysosomal damage response [8]. PLAA has been reproted to be associated with leukoencephalopathy [9]. Previous studies showed that PLAA was inactived in some cancers, including ovarian, lung, and breast cancer [10], and the application of PLAA polypeptide led to tumor regression in animal models of lung and breast cancer [11]. However, the role of PLAA in cancer metastasis is poorly known to date.

In the present study, we found that PLAA expression was significantly downregulated in ovarian cancer tissues, and patients with lower PLAA expression presented poorer prognosis. Functionally, PLAA suppressed migration and invasion of ovarian cancer cells and metastasis in the orthotopic xenograft mouse model. Mechanistically, PLAA inhibited METTL3 expression through the ubiquitin-mediated degradation and destabilized TRPC3 expression via METTL3-mediated m^6^A modification, which consequently suppressed intracellular calcium concentration and ovarian cancer metastasis. To the best of our knowledge, our findings is the first to demonstrate a suppressive role and the underlying mechanism of PLAA in ovarian cancer, which may provide a potential therapeutic approach for ovarian cancer metastasis.

## Results

### PLAA is downregulated in ovarian cancer and positively correlated with prognosis

**Fig. 1:**
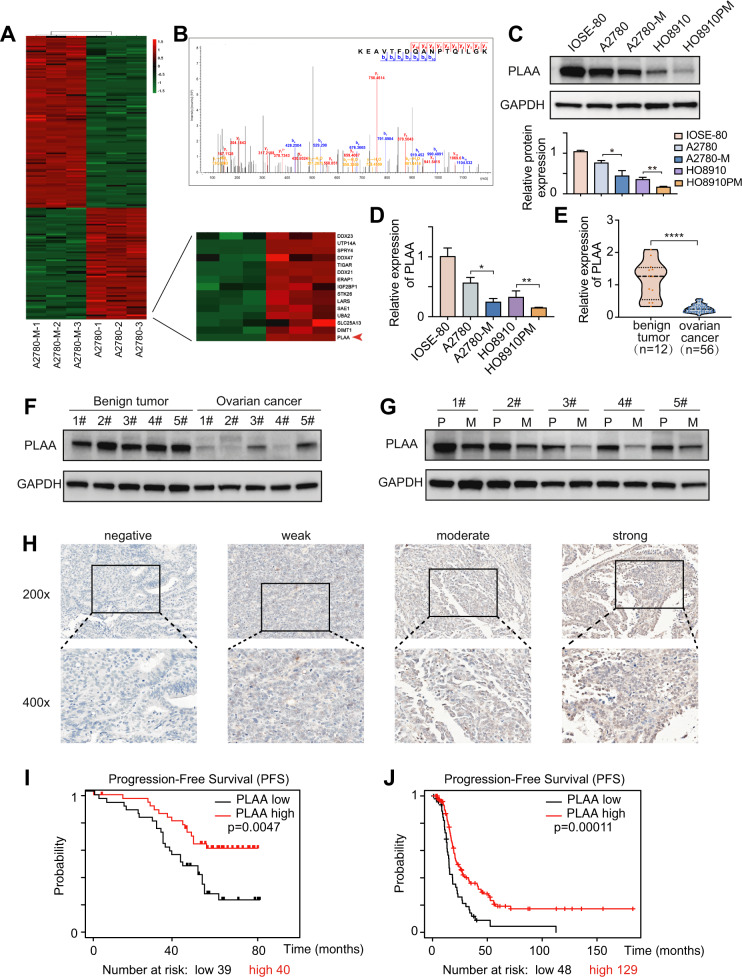
PLAA is downregulated in ovarian cancer and positively correlated with prognosis. **A** Differentially expressed proteins were compared between A2780-M and A2780 by using LC-MS/MS label-free quantitative proteomics. **B** A typical MS spectrum of PLAA. Immunoblot (**C**) and RT-qPCR analysis (**D**) of PLAA in IOSE-80 and two pairs of ovarian cancer cells with different metastatic ability. **E** RT-qPCR analysis of PLAA expression in 56 tissue samples of ovarian cancer and 12 tissue samples of ovarian benign tumor. **F** Immunoblot analysis of PLAA expression in ovarian cancer tissues and ovarian benign tumor tissues. **G** Immunoblot analysis of PLAA expression in ovarian cancer tissues and matched metastatic ovarian cancer tissues. P: primary ovarian cancer tissues. M: metastatic ovarian cancer tissues. **H** Representative IHC staining images of PLAA in ovarian cancer tissues (×200 and ×400 magnifications). Kaplan–Meier analysis of progression-free survival of 79 ovarian cancer patients in our hospital (**I**) and patients from TCGA data (**J**) with different PLAA expression. Data are representative of at least three independent experiments. *p < 0.05, **p < 0.01, ***p < 0.001, ****p < 0.0001.

A2780-M cell line with highly metastatic ability [5] and the parental cell line were selected for the protein expression profiles by liquid chromatography (LC)-mass spectrometry (MS)/MS label-free quantitative proteomics, and PLAA was identified as one of the most significantly downregulated proteins in highly metastatic cells (Fig. 1A). A typical MS spectrum of PLAA is shown in Fig. 1B. The endogenous expression of PLAA in ovarian cancer cell lines with different metastatic abilities, including A2780-M, A2780, HO8910PM, HO8910, and the normal ovarian epithelial cell line IOSE-80 were validated by western blot and RT-qPCR. The results showed that PLAA were lower in highly metastatic cell lines than those in parental cell lines, and were lower in ovarian cancer cells than those in normal ovarian cells (Fig. 1C, D). We additionally collected 56 fresh tissue samples of ovarian cancer and 12 fresh tissue samples of ovarian benign tumor for PLAA mRNA detection, and found PLAA mRNA was significantly lower in cancer tissues (Fig. 1E), especially reduced in patients with advanced stage than the early stage (Fig. S1A). Moreover, the same results were found in ovarian cancer from gene expression omnibus (GEO) (Fig. S1B) and TCGA datasets (Fig. S1C). In addition, PLAA protein expression were detected to be significantly lower in ovarian cancer tissues than that in benign tumor tissues (Fig. 1F; Fig. S1D). Interestingly, PLAA protein and mRNA expression was further decreased in metastatic ovarian cancer as compared with the primary ovarian cancer they were derived from (Fig. 1G; Fig. S1E).

To understand the clinical significance of PLAA, we retrospectively collected the clinicopathological data of 79 ovarian cancer patients and their formalin-fixed and paraffin-embedded tissue samples for immunohistochemistry of PLAA protein (Fig. 1H), and found the significant association of PLAA expression with FIGO stage, lymph node metastasis, and CA125 level in patients, as shown in Table 1 Kaplan–Meier survival analysis also revealed shorter progression-free survival (PFS) in patients with low PLAA expression than that in those with high PLAA expression (Fig. 1I). We additionally analyzed the data in TCGA database, and found the same results (Fig. 1J). Our data together suggest that patients with lower PLAA expression present shorter survival and PLAA may be a potential biomarker for predicting ovarian cancer prognosis.

**Table 1:** Clinical characteristics of 79 ovarian cancer patients depending on PLAA protein level.

variable	*N*	PLAA expression		*P* value
		Low	High	
Age (years)				
≤50	30	16	14	0.581
>50	49	23	26	
FIGO stage				
I/II	32	8	24	0.001**
III/IV	47	31	16	
Lymph node metastasis				
Negative	66	27	39	0.001**
Positive	13	12	1	
Ascitic fluid volume (ml)				
<500	51	22	29	0.135
≥500	28	17	11	
Serum CA125 (U/ml)				
<500	29	8	21	0.003**
≥500	50	31	19	

*Χ*^2^ Test was used to calculate the association between categorical variables. **p < 0.01.

### PLAA inhibits ovarian cancer migration and invasion in vitro and in vivo

**Fig. 2:**
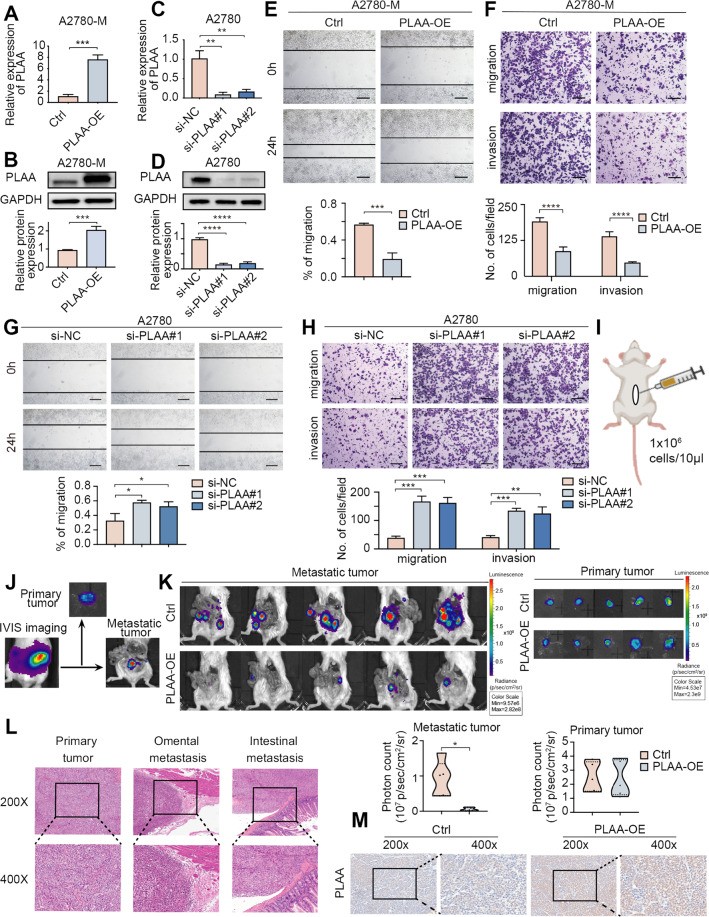
PLAA inhibits ovarian cancer migration and invasion in vitro and in vivo. A2780-M were transfected with PLAA-overexpressing plasmid or empty plasmid (**A**, **B**). A2780 cells were transfected with two PLAA siRNAs or negative control (**C**, **D**). PLAA expression was determined by RT-qPCR (**A**, **C**) or immunoblot analysis (**B**, **D**). A2780-M cells were transfected with PLAA-overexpressing plasmid or empty plasmid. Cellular migration and invasion were detected by wound healing (**E**) and transwell assay (**F**). Scale bar, 100 μm. A2780 cells were transfected with two PLAA siRNAs or negative control. Cellular migration and invasion were detected by wound healing (**G**) and transwell assay (**H**). Scale bar, 100 μm. **I** Schematic protocol for establishment of orthotopic ovarian cancer mouse model. **J** Representative images of orthotopic mouse model at the time of execution. Left, intact IVIS image; middle, image of dissected primary tumor; right, image of metastatic tumor. **K** Ovarian cancer cells were orthotopically transplanted into SCID mice. Representative bioluminescence images of dissected primary and metastatic ovarian cancer at the time of execution. The photon count indicated the tumor burden. **L** Histological features of primary, omental and intestinal metastatic lesions in the mouse model (×200 and ×400 magnifications). **M** Representative IHC staining images of PLAA in orthotopic xenograft model (×200 and ×400 magnifications). Data are representative of at least three independent experiments. *p < 0.05, **p < 0.01, ***p < 0.001, ****p < 0.0001.

To affirm the biological function of PLAA in ovarian cancer, PLAA-knockdown or PLAA-overexpressing cell lines were established (Fig. 2A–D; Fig. S2A–D). We found that PLAA overexpression dramatically inhibited cell migration and invasion (Fig. 2E, F; Fig. S2E, F), while PLAA downregulation promoted cell migration and invasion (Fig. 2G, H; Fig. S2G, H). However, PLAA had no effect on cell proliferation (Fig. S2I, J).

We next investigated the effect of PLAA on ovarian cancer metastasis in vivo utilizing the orthotopic xenograft model. PLAA-overexpressing or empty plasmid labeled with luciferase was stably transfected into A2780-M cells (named A2780-M-PLAA-luc cells or A2780-M-control-luc cells). The left ovary of each SCID mouse was pulled out from the median abdominal incision and A2780-M-PLAA-luc or A2780-M-control-luc cells were orthotopically injected into the ovary sub epithelium (each group = 5) (Fig. 2I). In vivo imaging system (IVIS) was performed every week to monitor tumor progression, and mice were euthanized after 6 weeks. The primary and metastatic tumors were separately assessed via taking off the primary tumors (Fig. 2J). IVIS scan showed that PLAA overexpression significantly inhibited metastasis in the xenograft model compared with controls, without changing their primary tumors (Fig. 2K). Hematoxylin and eosin (H&E) staining displayed the typical morphology of primary tumor, omental, and intestinal metastasis of ovarian cancer (Fig. 2L). Immunohistochemistry staining assay showed the overexpression efficiency of PLAA in orthotopic xenograft model (Fig. 2M). Our results together demonstrate that PLAA acts as a tumor metastatic suppressor in ovarian cancer.

### TRPC3 is negatively correlated with PLAA expression

**Fig. 3:**
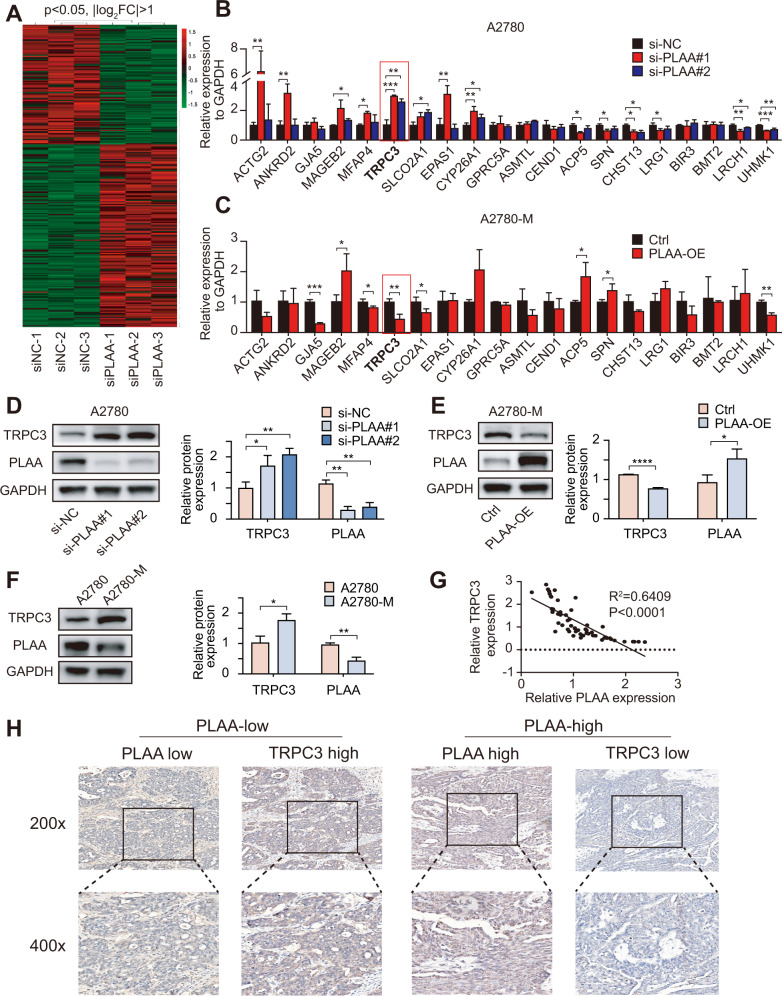
TRPC3 is negatively correlated with PLAA expression. **A** The heat map of differentially expressed mRNAs between A2780 cells with PLAA knockdown and without. RT-qPCR analysis of differentially expressed mRNAs in A2780 cells transfected with PLAA siRNAs (**B**) and A2780-M cells transfected with PLAA plasmids (**C**). The red frame shows TRPC3. **D** Immunoblot analysis of TRPC3 protein levels between A2780 cells with PLAA knockdown and without. **E** Immunoblot analysis of TRPC3 protein levels between A2780-M cells with PLAA overexpression and without. **F** Immunoblot analysis of PLAA and TRPC3 expression in A2780-M and A2780 cells. **G** RT-qPCR analysis of PLAA and TRPC3 expression in 56 ovarian tumor tissues. **H** Representative IHC staining images of PLAA and TRPC3 in ovarian cancer tissues (×200 and ×400 magnifications). Data are representative of at least three independent experiments. *p < 0.05, **p < 0.01, ***p < 0.001, ****p < 0.0001.

To understand the underlying mechanism by which PLAA inhibits ovarian cancer metastasis, transcriptome sequencing was performed in A2780 cells with PLAA knockdown and without, respectively. Hierarchical clustering indicated 165 upregulated genes and 109 downregulated genes (|log_2_ fold change| > 1, *p* < 0.05) in cells with PLAA knockdown (Fig. 3A). We selected ten top upregulated and ten top downregulated genes to validate their expression in A2780 and HO8910 cells with PLAA knockdown or in A2780-M and HO8910PM cells with PLAA overexpression, and found that in those differentially expressed genes, TRPC3 was the only one that presented consistently upregulated expression in both cells and by both PLAA siRNAs (Figs. 3B; S3A). On the contrary, TRPC3 was downregulated in A2780-M and HO8910PM cells with PLAA overexpression (Figs. 3C; S3B). In addition, TRPC3 protein expression was validated to be upregulated with PLAA knockdown (Fig. 3D; S3C), and to be downregulated with PLAA overexpression (Figs. 3E; S3D), as well as in highly metastatic cells comparing to their parental cells (Figs. 3F; S3E). We further found a significant negative correlation of the expression between PLAA and TRPC3 mRNA in 56 cancer samples (Fig. 3G), and IHC staining assay also showed the negative correlation between PLAA and TRPC3 (Fig. 3H). Therefore, these observations validate that TRPC3 probably is the potential target of PLAA in ovarian cancer.

### TRPC3 behaves as an oncogene and acts as a downstream target of PLAA

**Fig. 4:**
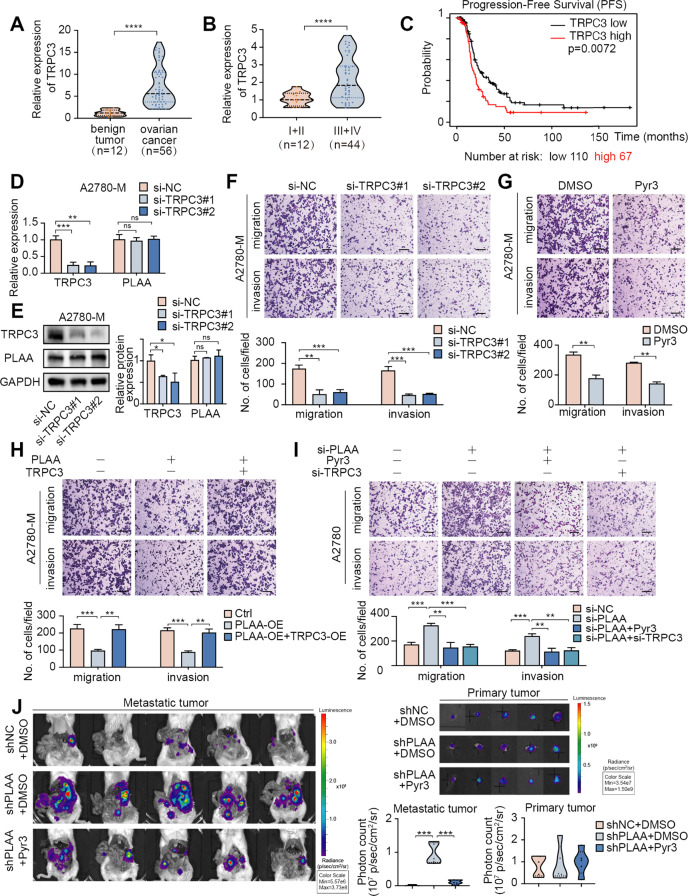
TRPC3 behaves as an oncogene and acts as a downstream target of PLAA. **A** RT-qPCR analysis of TRPC3 expression in 56 tissue samples of ovarian cancer and 12 tissue samples of ovarian benign tumor. **B** RT-qPCR analysis of TRPC3 expression in ovarian cancer tissues of late stages and early stages. **C** Kaplan–Meier analysis of progression-free survival of patients from TCGA data with different TRPC3 expression. A2780-M cells were transfected with two TRPC3 siRNAs or negative control. TRPC3 expression was determined by RT-qPCR (**D**) or immunoblot analysis (**E**). **F** A2780-M cells were transfected with two TPRC3 siRNAs or negative control. Cellular migration and invasion were detected by transwell assay. Scale bar, 100 μm. **G** Cellular migration and invasion of A2780-M cells treated with Pry3 or DMSO were detected by transwell assay. Scale bar, 100 μm. **H**, **I** A2780-M cells were transfected with PLAA plasmid, PLAA plasmid plus TRPC3 plasmid, and negative control, respectively (**H**). A2780 cells were transfected with PLAA siRNA, PLAA siRNA plus TRPC3 siRNA, PLAA siRNA plus Pyr3 treatment (2 μM, 24 h), and negative control, respectively (**I**). Cellular migration and invasion were detected by transwell assay. Scale bar, 100 μm. **J** Ovarian cancer cells were orthotopically transplanted into SCID mice, and mice were intravenously treated with DMSO or Pyr3 (5 mg/kg) for twice a week from the first week after implantation. Representative bioluminescence images of dissected primary and metastatic ovarian cancer at the time of execution. The photon count indicated the tumor burden. Data are representative of at least three independent experiments. *p < 0.05, **p < 0.01, ***p < 0.001, ****p < 0.0001, ns no significant.

To further characterize the role of TRPC3 in ovarian cancer, we first detected the expression of TRPC3 in our clinical ovarian cancer samples. We validated that TRPC3 mRNA were dramatically upregulated in ovarian cancer tissues compared with benign ovarian tumor tissues (Fig. 4A), and were higher in late stages of ovarian cancer tissues compared with the early stages (Fig. 4B). Furthermore, a Kaplan–Meier survival analysis of TCGA database showed that higher TRPC3 expression is correlated with shorter progression-free survival (PFS) (Fig. 4C). Thus, we established TRPC3-knockdown or TRPC3-overexpressing cell models according to the basic TRPC3 level, and found that PLAA expression remained unaltered whether TRPC3 was knocked down or over expressed (Figs. 4D, E; S4A–D), but transwell assay revealed that TRPC3 knockdown or treatment with Pyr3, an inhibitor of TRPC3, inhibited migration and invasion in A2780-M and HO8910PM cells (Figs. 4F, G; S4E, F), whereas TRPC3 overexpression presented an opposite effect (Fig. S4G).

Moreover, we observed that migration and invasion ability decreased by PLAA overexpression were retrieved by TRPC3 overexpression (Figs. 4H; S4H). Conversely, migration and invasion ability accelerated by PLAA knockdown were abrogated by TRPC3 siRNA or treatment with Pyr3 (Figs. 4I; S4I). Utilizing the orthotopic ovarian cancer mouse model again, we injected A2780-shNC-luc (*n* = 5) or A2780-shPLAA-luc cells into ovaries of mice (*n* = 10), and randomly divided A2780-shPLAA-luc group into two sub-groups (each sub-group = 5) at day 7 after implantation (Fig. S4J). Pyr3 or DMSO was intravenously injected twice a week from week 1 for 8 times, and then the mice were euthanized after 6 weeks. We found that the mice injected with A2780-shPLAA-luc, compared with the control group, showed wider metastasis, which was significantly abolished by Pyr3 treatment, but no significant changes were detected in primary tumors (Fig. 4J). Our results together suggest that PLAA inhibits ovarian cancer metastasis in a TRPC3 dependent manner.

### PLAA inhibits ovarian cancer metastasis by attenuating TRPC3-mediated intracellular calcium level

**Fig. 5:**
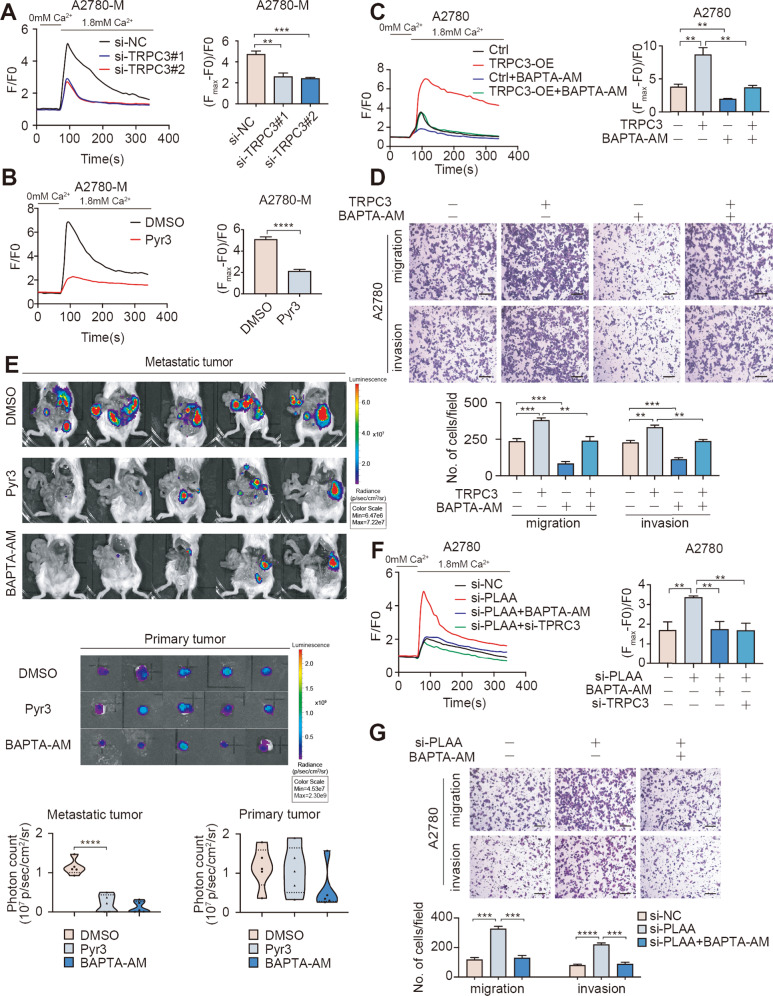
PLAA inhibits ovarian cancer metastasis by attenuating TRPC3-mediated intracellular calcium level. A2780-M cells were transfected with two TRPC3 siRNAs (**A**) or treated with Pyr3 (**B**). Addition of external calcium(1.8 mM) led to an increase in fluorescence intensity and representative Ca^2+^ images tracing reflected changes of calcium concentration in the cytoplasm. F/F0: fluorescence normalized to baseline fluorescence. The net change in Ca^2+^ levels was normalized to (*F*_max_ − *F*_0_)/*F*_0_. **C** A2780 cells transfected with TRPC3-overexpressing plasmid or empty plasmid were treated with BAPTA-AM (20 μg/mL, 24 h) or not. Addition of external calcium(1.8 mM) led to an increase in fluorescence intensity and representative Ca^2+^ images tracing reflected changes of calcium concentration in the cytoplasm. F/F0: fluorescence normalized to baseline fluorescence. The net change in Ca^2+^ levels was normalized to (*F*_max_ − *F*_0_)/*F*_0_. **D** A2780 cells transfected with TRPC3-overexpressing plasmid or empty plasmid were treated with BAPTA-AM (20 μg/mL, 24 h) or not. Cellular migration and invasion were detected by transwell assay. Scale bar, 100 μm. **E** Ovarian cancer cells were orthotopically transplanted into SCID mice, and mice were intravenously treated with Pyr3 (5 mg/kg), BAPTA-AM (5 mg/kg) or DMSO for twice a week from the first week after implantation. Representative bioluminescence images of dissected primary and peritoneal metastatic ovarian cancer at the time of execution. The photon count indicated the tumor burden. **F** A2780 cells transfected with PLAA siRNA, PLAA siRNA plus BAPTA-AM (20 μg/mL, 24 h) treatment, PLAA siRNA plus TRPC3 siRNA, and negative control, respectively. Addition of external calcium(1.8 mM) led to an increase in fluorescence intensity and representative Ca^2+^ images tracing reflected changes of calcium concentration in the cytoplasm. F/F0: fluorescence normalized to baseline fluorescence. The net change in Ca^2+^ levels was normalized to (*F*_max_ − *F*_0_)/*F*_0_. **G** A2780 cells transfected with PLAA siRNA, PLAA siRNA plus BAPTA-AM (20 μg/mL, 24 h) treatment, and negative control, respectively. Cellular migration and invasion were detected by transwell assay. Scale bar, 100 μm. Data are representative of at least three independent experiments. *p < 0.05, **p < 0.01, ***p < 0.001, ****p < 0.0001.

TRPC3 is a high calcium permeability cationic channel that governs the calcium-related signaling [12]. Intracellular calcium, a versatile second messenger, participates in multiple biological behaviors, such as proliferation and metastasis in cancer cells [13, 14]. In the presence of external solution containing 1.8 mM free calcium, we observed that the intracellular Ca^2+^ level was decreased when TRPC3 was knocked down or treated with Pyr3 (Figs. 5A, B; S5A, B). On the contrary, intracellular Ca^2+^ level was increased when TRPC3 was overexpression and the increased Ca^2+^ level was retrieved by the treatment of BAPTA-AM, a well-recognized intracellular calcium chelator (Figs. 5C; S5C). We further observed the effect of Ca^2+^ influx on TRPC3 promoting ovarian cancer metastasis. As expected, we found that migration and invasion accelerated by TRPC3 overexpression were abrogated in A2780 and HO8910 cells treated by BAPTA-AM (Figs. 5D; S5D). Further, we employed the orthotopic mouse model. Mice were injected with A2780-M-luc (n = 15) cells and were randomly divided into three group (each group = 5) at day 7 after implantation (Fig. S5E), Pyr3, BAPTA-AM or DMSO was intravenously injected twice a week from week 1 for 8 times, and mice were euthanized after 6 weeks. Hematoxylin and eosin staining for the major organs showed that Pyr3 and BAPTA-AM had no significant adverse effects under our experimental condition (Fig. S5F). IVIS scan showed that metastasis was significantly inhibited in the xenograft model with Pyr3 or BAPTA-AM treatment compared with controls, but no significant changes were detected in primary tumors (Fig. 5E).

In addition, in the presence of external solution containing 1.8 mM free calcium, we found that TRPC3 downregulation or BAPTA-AM treatment dramatically decreased the level of intracellular Ca^2+^ elevated by PLAA downregulation (Figs. 5F; S5G). As expected, the migration and invasion accelerated by PLAA downregulation were also rescued by BAPTA-AM (Figs. 5G; S5H). Our results collectively demonstrate that PLAA inhibits ovarian cancer metastasis via downregulating TRPC3-mediated intracellular calcium level.

### PLAA destabilizes TRPC3 mRNA through m^6^A modification

**Fig. 6:**
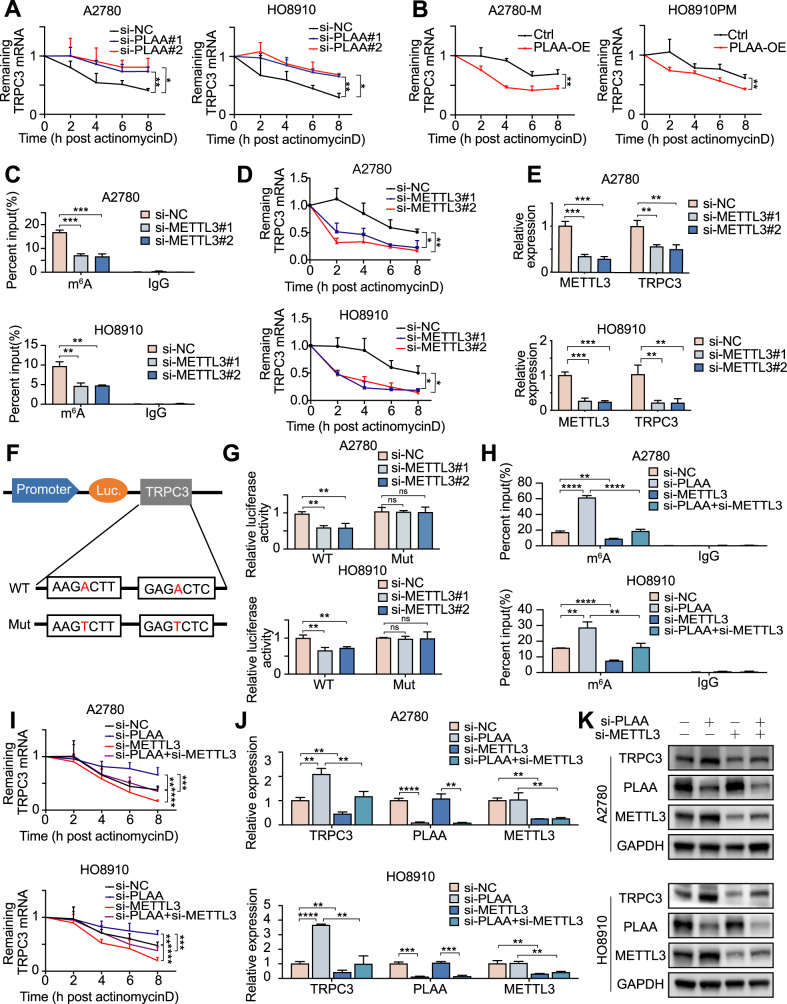
PLAA destabilizes TRPC3 mRNA through m^6^A modification. PLAA-knockdown (**A**) and PLAA-overexpressing (**B**) cells were treated with actinomycin D (5 μg/ml) for different hours. TRPC3 mRNA expression was detected by RT-qPCR analysis (normalized to 0 h). A2780 and HO8910 cells were transfected with METTL3 siRNA or negative control, respectively. m^6^A modification of TRPC3 was detected by MeRIP-qPCR analysis using anti-IgG and anti-m^6^A antibodies (**C**), the RNA decay rate was detected after treatment with actinomycin D (normalized to 0 h) (**D**), TRPC3 and METTL3 expressions were determined by RT-qPCR (**E**). **F** Wild or m^6^A motif mutant TRPC3 sequence was fused with firefly and renilla luciferase reporter. Relative luciferase activity was calculated by normalizing to renilla activity. **G** Relative luciferase activity was measured in METTL3-downregulated cells transfected with wild or mutant TRPC3 plasmid. A2780 and HO8910 cells were transfected with PLAA siRNA, METTL3 siRNA, PLAA siRNA plus METTL3 siRNA, and negative control, respectively. m^6^A modification of TRPC3 was detected by MeRIP-qPCR analysis (**H**), the RNA decay rate was detected after treatment with actinomycin D (normalized to 0 h) (**I**), TRPC3, PLAA, and METTL3 expression were determined by RT-qPCR (**J**) and immunoblot analysis (**K**), respectively. Data are representative of at least three independent experiments. *p < 0.05, **p < 0.01, ***p < 0.001, ****p < 0.0001, ns no significant.

As we stated above, PLAA can directly regulate TRPC3 mRNA level. Here, we found that inhibiting the synthesis of mRNA using actinomycin D, a commonly used transcription inhibitor, cannot rescue the upregulated TRPC3 mRNA induced by PLAA knockdown, indicating that it was in a transcription-independent pathway (Fig. S6A). Meanwhile, we found that PLAA knockdown significantly increased the stability of TRPC3 mRNA (Fig. 6A), whereas PLAA overexpression exerted an opposite role (Fig. 6B), suggesting that mRNA stability change is the main cause of TRPC3 upregulation in the PLAA downregulated group.

Considering that m^6^A modification is a common regulation model of mRNA at the post-transcriptional level, including mRNA stability [15]. First, we regulated m^6^A modification by modulating the expression of METTL3, the main methyltransferase of m^6^A modification. We knocked down METTL3, and interestingly found remarkably reduced TRPC3 m^6^A modification level (Fig. 6C), mRNA stability (Fig. 6D), mRNA level (Fig. 6E), and protein level (Fig. S6B) in A2780 and HO8910 cells. To further demonstrate the essential role of m^6^A in the regulation of TRPC3, we replaced N^6^-methylated adenosine (A) with thymine (T) in the m^6^A consensus sequence of TRPC3 mRNA to establish a mutant TRPC3 that resists m^6^A modification (Fig. 6F), and found that the luciferase activity was decreased in cells transfected with TRPC3-WT plasmid, but not mutant ones, when METTL3 was silenced (Fig. 6G).

Further, to investigate whether PLAA mediates TRPC3 by m^6^A modification, we carried out a rescue assay, and found that the elevated m^6^A modification level and the prolonged half-life of TRPC3 mRNA induced by PLAA depletion was reversed by METTL3 knockdown (Fig. 6H, I). Moreover, the elevation of TRPC3 mRNA and protein level induced by PLAA knockdown was also retrieved by attenuation of METTL3 (Figs. 6J, K; S6C). Our results together indicate that PLAA regulates the expression of TRPC3 mRNA via m^6^A modification.

### PLAA promotes METTL3 degradation via the ubiquitin-mediated pathway in ovarian cancer

**Fig. 7:**
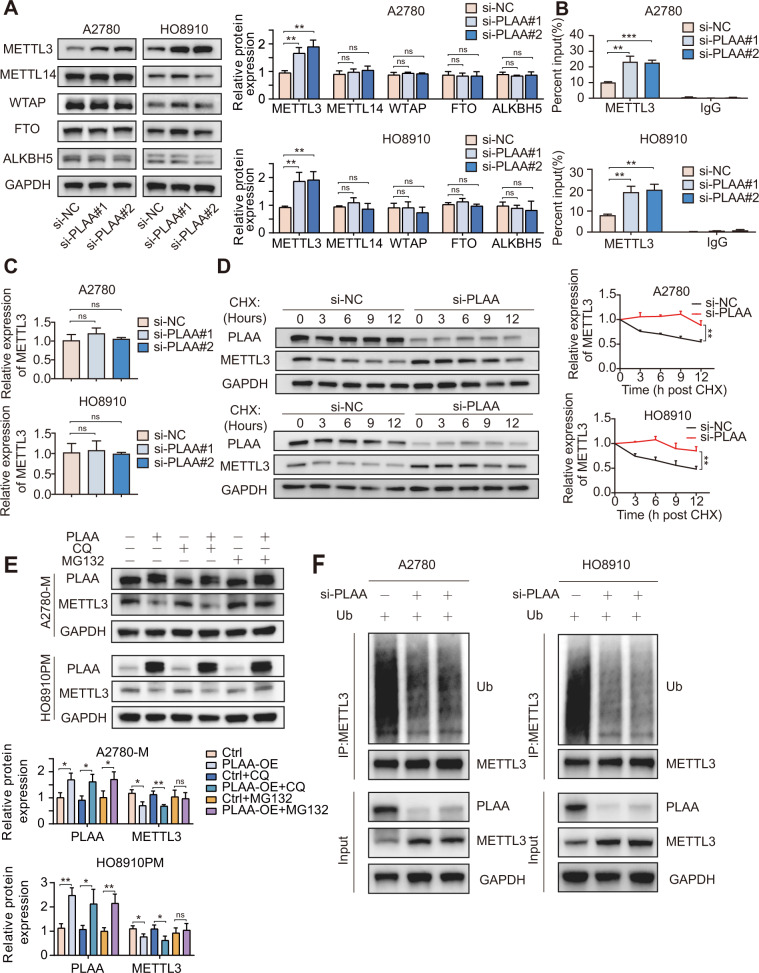
PLAA promotes METTL3 degradation via the ubiquitin-mediated pathway in ovarian cancer. **A** Expression of m^6^A writers (METTL3, METTL14, and WTAP) and erasers (ALKBH5 and FTO) were determined by immunoblot analysis. **B** RNA immunoprecipitation assays in PLAA-knockdown cells using METTL3 antibody to enrich TRPC3. The enrichment was calculated as input %. **C** A2780 and HO8910 cells were transfected with PLAA siRNAs or negative control, and METTL3 expression was determined by RT-qPCR. **D** PLAA-knockdown cells were treated with 50 μg/ml of cycloheximide (CHX) for different hours. PLAA and METTL3 expressions were detected by immunoblot analysis. **E** A2780-M and HO8910PM cells with or without PLAA overexpression were treated with 40 μM MG132 or 20 μM chloroquine (CQ) for 8 h. PLAA and METTL3 expressions were detected by immunoblot analysis. **F** A2780 and HO8910 cells were transfected with ubiquitin (Ub) and PLAA siRNA. Cell lysates were immunoprecipitated with METTL3 antibody followed by immunoblot analysis with ubiquitin antibody. Data are representative of at least three independent experiments. *p < 0.05, **p < 0.01, ***p < 0.001, ****p < 0.0001, ns no significant.

Since PLAA regulates the expression of TRPC3 mRNA by m^6^A modification, we detected m^6^A modification related proteins, including writers (m^6^A methyltransferases, including METTL3, METTL14 and WTAP) and erasers (m^6^A demethylases, including FTO and ALKBH5). As expected, we found that METTL3, but not others, was remarkably elevated in cells with PLAA knockdown (Fig. 7A), suggesting that TRPC3 mRNA was regulated by METTL3. In addition, RNA immunoprecipitation assay showed that METTL3-specific antibody dramatically enriched TRPC3 mRNA, compared to the IgG control, while PLAA knockdown significantly intensified the enrichment of TRPC3 mRNA in A2780 and HO8910 cells (Fig. 7B). Considering that PLAA knockdown had no effect on the mRNA level of METTL3 (Fig. 7C), we suggested changes at protein level may be the main reason for METTL3 upregulation in the PLAA downregulated group. Thus, we treated A2780 and HO8910 cells with cycloheximide (CHX) to inhibit protein biosynthesis, and found a significantly extended half-life of METTL3 protein in cells with PLAA knockdown (Fig. 7D). We further observed the restored METTL3 protein expression in A2780-M and HO8910PM cells with PLAA overexpression after the treatment of proteasome inhibitor MG132, but not lysosomal autophagy inhibitor chloroquine (CQ) (Fig. 7E). These results suggested that METTL3 was degraded via the ubiquitin-mediated degradation pathway. Thus, we performed the ubiquitination experiment, and found that PLAA downregulation reduced ubiquitination of METTL3 in A2780 and HO8910 cells as expected (Fig. 7F). These results suggest that PLAA inhibits METTL3 protein expression via the ubiquitin-mediated degradation pathway.

## Discussion

Previous studies revealed that PLAA inactivity was associated with ovarian and other cancers [10, 11]. In this study, we observed that PLAA expression was significantly downregulated in two highly metastatic ovarian cancer cells than that in lower metastatic cells, and in ovarian cancer tissues compared to ovarian benign tumor tissues. In addition, PLAA expression was decreased in metastatic ovarian cancer tissues as compared with the primary ovarian cancer tissues that they were derived from. Although the reason for the decline of PLAA expression is not clear to date, our in vitro and in vivo experiments further showed that the PLAA upregulation inhibited migration and invasion of ovarian cancer cells, conversely, PLAA downregulation promoted migration and invasion of ovarian cancer cells. PLAA overexpression also suppressed metastasis in the mouse model of orthotopic xenograft. Importantly, we found that patients with lower PLAA expression were associated with high-risk clinicopathological features, such as FIGO stage, lymph node metastasis and CA125 level, and presented shorter survival. All our findings suggest that PLAA acts as a tumor suppressor in ovarian cancer metastasis, and PLAA may have a potential to be clinically used as a biomarker associated with patient prognosis and a therapeutic target in metastatic ovarian cancer.

The mechanism by which PLAA functions is unknown so far. We utilized RNA-seq to identify candidates of PLAA downstream targets, and found TRPC3 to be upregulated in ovarian cancer cells with PLAA knockdown and there was a reverse correlation between PLAA and TRPC3 expression in ovarian cancer cells and tissues. Previous studies showed that TRPC3 was a member of the transient receptor potential (TRP) channel superfamily [16, 17] and crucial for the level of calcium concentration [18]. Calcium ion, as one of the most important second messengers in intracellular signaling network, regulates lots of physiopathological processes, such as metastasis in cancer cells [19]. Increasing evidence has demonstrated that TRPC3 is involved in a series of physiological activities [12, 20] and plays an important role in tumor proliferation, invasion and migration, including lung cancer [21], gastric cancer [22], bladder cancer [23] and ovarian cancer [24]. Here, we also found that TRPC3 knockdown inhibited migration and invasion in ovarian cancer cells, whereas TRPC3 overexpression presented the opposite effect. We further validated that TRPC3 functioned through regulating intracellular Ca^2+^ level. Importantly, TRPC3 silencing or an inhibitor of TRPC3 (Pyr3) treatment dramatically reversed increased migration and invasion of ovarian cancer cells and metastasis of xenograft induced by PLAA downregulation, suggesting that TRPC3 is a downstream target of PLAA, participating in PLAA mediating ovarian cancer metastasis. Previous studies also found that Ca^2+^ channel blockers, like diltiazem and verapamil, significantly reduce the invasive properties of patient-derived spheroid cultures and invasion of squamous cell carcinoma in the mouse model [25]. Combined with previous report, our findings suggest that calcium channel inhibitors may have a clinical potential to be used in the suppression of ovarian cancer metastasis through PLAA-TRPC3 pathway.

It has been known that m^6^A modification plays an important role in RNA stability and production [15]. N6-methyladenosine (m^6^A), as the most common modification of eukaryotic mRNA [26], has attracted much attention. The methylation of m^6^A is regulated by “writers” (methyltransferases) and “erasers” (demethylases) [27]. The classical “writers” are comprised of methyltransferase-like 3 (METTL3), methyltransferase-like 14 (METTL14) [28] and Wilms tumor 1 associated protein (WTAP) [29], while the classical “erasers” include fat mass and obesity-associated (FTO) [30] and AlkB homolog 5 (ALKBH5) [31]. Recent studies have revealed the association of dysregulation of m^6^A modification with various cancers, including bladder [32], liver [33], ovarian [34], and other cancers. In this study, we found that PLAA downregulation still elevated TRPC3 mRNA level in the presence of transcription inhibitor, suggesting that the regulation of TRPC3 by PLAA does not occur at the transcriptional level. Therefore, we carried out the mRNA stability assay and found that PLAA knockdown remarkably elevated the TRPC3 mRNA stability. Thus, screening of candidates associated with m^6^A modification in PLAA regulating TRPC3 was performed, and METTL3 was only upregulated protein among 3 “writers” and 2 “erasers” when PLAA knockdown. METTL3 and METTL14 form a stable heterodimer to affect the metastatic potential of cancer cells via distinct mechanism [35, 36], for example, METTL3 promoted the initiation and metastasis of ovarian cancer by inhibiting CCNG2 expression [37]. We also identified that METTL3 downregulation neutralized the increased TRPC3 m^6^A modification level, mRNA stability, mRNA level, and protein level induced by PLAA knockdown, suggesting that m^6^A modification contributes to PLAA regulating TRPC3 mRNA level. The m^6^A modification can result in different biologic process through different reader proteins. Previous study has revealed that IGF2BPs contribute to mRNA stability [38] and IGF2BP1 and IGF2BP3 are associated with poor disease outcome in ovarian cancer [39, 40]. Thus, protein IGF2BPs are possible to be potential readers in TRPC3 m^6^A modification.

Another function of PLAA is regulation of protein ubiquitination that is a common and dynamic post-translational modification in nearly all eukaryotic biology [41]. Previous studies have demonstrated that PLAA targets ubiquitinated proteins and degrades through the ubiquitin-proteasome system [7, 42]. In this study, we found that PLAA affected the protein level of METTL3, but not mRNA level. Our ubiquitination assay showed that downregulation of PLAA was accompanied by reduced ubiquitination of METTL3. However, the detailed mechanism requires further investigation.

**Fig. 8:**
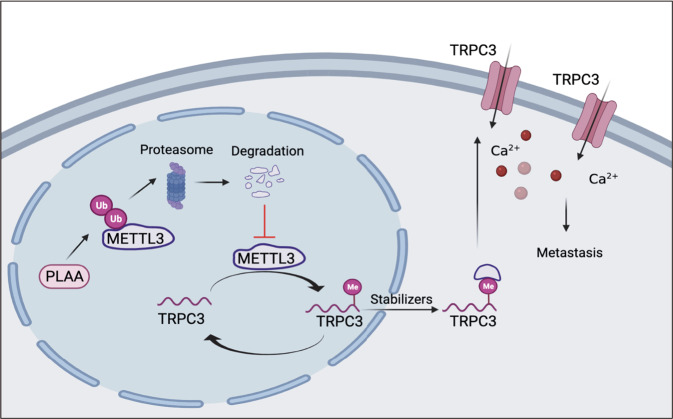
The mechanistic illustration showed PLAA-METTL3-TRPC3 axis in ovarian cancer. In brief, PLAA degrades METTL3 by the ubiquitin-mediated degradation pathway, consequently leading to the destabilization of m^6^A-modified mRNAs, including the oncogenic gene TRPC3 which modulates intracellular calcium flux, which, therefore, restrains metastasis of ovarian cancer cells.

In summary, our study showed the association of downregulated PLAA with ovarian cancer metastasis and poorer prognosis of patients, PLAA inhibits migration and invasion of ovarian cancer cells via regulating TRPC3 and intracellular calcium level, and suppressed metastasis of orthotopic xenograft in mouse model. PLAA downregulates METTL3 via proteasome degradation pathway, and the METTL3-mediated m^6^A modification contributes to PLAA modulating TRPC3 mRNA (Fig. 8). Our findings demonstrate a suppressive role, as well as underlying mechanism, of PLAA in ovarian cancer metastasis, which may provide a potential approach to block ovarian cancer metastasis in clinic.

## Materials and methods

### Patient specimens

This study was approved by the Ethics Committee of Women’s Hospital, School of Medicine, Zhejiang University (granted number: IRB-20210147-R). All tissue samples were gathered from our hospital and informed consent was obtained from each patient before surgery. No patients underwent radiotherapy or chemotherapy before surgery. The patient information was listed in Table S1. All pathological diagnosis was re-reviewed by an expert pathologist.

### Animal studies

All animal experiments were approved by Animal Ethical and Welfare Committee of Zhejiang Chinese Medical University (granted number: IACUC-20191216-02). Female severe combined immunodeficiency (SCID) mice, aged 4–6 weeks, were used as the orthotopic xenografts model. Mice were anesthetized by peritoneal injection of 0.3% pentobarbital (0.25 ml/10 g), the luciferase-expressed cells (1 × 10^6^ cells in 10 μl PBS) were injected into the left ovary that was pulled out from the small abdominal midline incision, and the bio-gel was applied for preventing cells leaking into the abdominal cavity. Tumor development and metastasis were monitored once a week by using the In Vivo Imaging System (IVIS) Lumina LT system (PerkinElmer, USA) after peritoneal injection of 150 mg/kg D-luciferin. Seven days after transplantation, bioluminescence intensity was applied to randomize mice into groups with different treatments to make sure similar level of bioluminescence in each group. To accurately assess the metastatic tumors, the primary tumors were dissected and the photon values of metastatic tumors were obtained. Dissected tissues were quickly fixed in 4% paraformaldehyde and embedded in paraffin for Hematoxylin and eosin (H&E) and immunohistochemistry (IHC) staining.

### Cell culture

The human epithelial ovarian cancer cell lines A2780 and A2780-M cell lines were kindly gifted by the Cancer Research Institute, Zhejiang Cancer hospital, China. HO8910 and HO8910PM were obtained as described previously [43]. The normal ovarian epithelial cell line, IOSE-80, was kindly gifted by Prof. Lu Yan, Zhejiang University. These cells were authenticated by DNA(STR) profiling. Cells were incubated at 37 °C in a 5% CO_2_ incubator with humidified environment, and cultured with RPMI-1640 medium (BasalMedia, China), which were routinely supplemented with 10% fetal bovine serum (Everyday Green, China), penicillin (100 units/ml) and streptomycin (100 μg/ml).

### LC–MS/MS label-free quantitative proteomics

Ovarian cancer cells (three independent samples in each group) were cracked in 300 μL lysis buffer, sonicated and centrifuged at 12,000 × *g* for 10 min. The supernatant was then collected, and measured the concentration by Bicinchoninic Acid Assay. The 10 μg proteins of each sample were acquired and separated by 12% SDS-PAGE gel. Other protein extracts were incubated for digestion at 37 °C for 16 h with Lys-C/Trypsin. The digested peptides were desalted by C18-Reverse-Phase SPE Column, and then the column was washed to elute the peptides by 0.1% trifluoroacetic acid. MS analysis was performed by a Q-Exactive mass spectrometer (Thermo scientific, USA). Full MS scans were acquired in the mass range of 350–1500 m/z. The ten most intense peaks in MS were fragmented with higher-energy collisional dissociation (HCD) with NCE of 32. Peaks lists were generated from raw data files and search against Human Protein Database using MaxQuant. Differential protein expression analysis was done using the R package DEseq2, *P* value < 0.05 and log2 | fold change| > 1 was set as the threshold for significantly differential expression.

### Lentiviruses, plasmids, and siRNAs transfection

PLAA overexpression lentivirus, PLAA shRNA lentiviruses, and negative control lentiviruses were synthesized by Genechem (China) and transduced into cells according to the manufacturer’s instructions. PLAA, TRPC3, and ubiquitin overexpression plasmids were constructed with the GV492, GV657, GV712 vector respectively by Genechem (China) and transfected into cells using X-treme GENE HP DNA Transfection Reagent (Roche, China). PLAA, TRPC3, METTL3 siRNA, and control siRNA were synthesized by Genepharma (China). Transient transfection was performed by DharmaFECT Transfection Reagents (Thermo, USA) in accordance with the standard protocol. The target sequences of shRNAs and siRNAs were listed in Table S2.

### RNA extraction and RT-qPCR analysis

Total RNA of cells was extracted by TRIzol Reagent (Invitrogen, USA). RNA was reverse transcribed by PrimeScript RT reagent Kit with gDNA eraser (Takara, Japan). PCR analysis was conducted using TB Green Premix Ex Taq (Takara, Japan) and 7900HT fast real-time PCR system (Life Technologies, USA). Primer sequences are listed in Table S3. The relative mRNA expressions were calculated by the 2^−ΔΔCt^ method normalized to GAPDH.

### RNA sequencing

A2780 cells were transfected according to manufacturer’s protocol. The total RNA was extracted by TRIzol Reagent (Invitrogen, USA). RNA purity and quantification were evaluated using the NanoDrop 2000 spectrophotometer (Thermo Scientific, USA). RNA integrity was assessed using the Agilent 2100 Bioanalyzer (Agilent Technologies, USA). The samples with RNA Integrity Number (RIN) ≥ 7 were used for library construction by using TruSeq Stranded mRNA LTSample Prep Kit (llumina, USA) according to the manufacturer’s instructions. And these libraries were sequenced on an Illumina HiSeq X Ten sequencing platform by OE Biotech. Co., Ltd (China). Differential gene expression analysis was done using the R package DEseq2, *P* value < 0.05 and log_2_|fold change| > 1 was set as the threshold for significantly differential expression. Hierarchical cluster analysis of DEGs was performed to demonstrate the expression pattern of genes in different groups and samples.

### Western blot analysis and immunohistochemistry

For western blot analysis, cells were lysed with RIPA, and protein samples were separated by 10% SurePAGE gels (GenScript, USA) and transferred to 0.22 μm PVDF membranes (Bio-Rad, USA) using the eBlot L1 protein transfer system (GenScript, USA). At room temperature, 5% milk was used to block the membrane. Membrane was exposed to ImageQuant LAS 4000 mini (Cytiva, Japan).

For immunohistochemistry analysis, the formalin-fixed and paraffin-embedded samples were first deparaffinized and rehydrated, followed by PBS washing. Antigen retrieval was performed in 0.01 M sodium citrate buffer (pH 6.0) at 100 °C for 15 min. The positive cells were scored as: 1 for 0–25%, 2 for 26–50%, 3 for 51–75% and 4 for 76–100%. Staining intensity was scored as: 1 for negative, 2 for weak, 3 for moderate, and 4 for strong staining. The score for each microscopic field was obtained by multiplying the two parts of score, the sum was acquired by adding the scores of the five microscopic fields. The antibodies used were listed in Table S4.

### Transwell assay and wound healing assay

The 24-well transwells (8 μm pore size, Falcon, USA) were used for evaluating cell invasion and migration ability. A total of 1 × 10^5^/200 μL cells were cultured in serum-free medium in the upper chambers, with or without Matrigel (Corning, USA), and medium containing 10% FBS was added into the bottom chambers. The non-invasive or non-migratory cells were removed after 12–24 h, penetrated cells were fixed, stained, and counted.

A total of 3 × 10^4^ cells were suspended in 70 μL of RPMI-1640 medium and were seeded in ibidi culture insert (ibidi, Germany). The incubation chambers were placed in a 6-well plate. When cells were at full confluency, the insert was removed to create a gap, and serum-free medium was subsequently added. Image was captured by microscope at 0 h and 24 h, the distance between the gap was measured, the data were collected from three independent experiments and the migration rate of the cells was expressed as relative gap closure by using the ImageJ software.

### RNA decay assay

To evaluate the RNA stability, the culture medium was supplemented with 5 μg/mL actinomycin D (Sigma-Aldrich, USA), then cells were collected at indicated different time points. Total RNA was isolated and subjected to RT-qPCR subsequently to analyze the relative abundance of TRPC3 mRNA (relative to 0 h).

### MeRIP-qPCR

m^6^A modifications of individual genes were determined using MeRIP-qPCR assay. RNA was isolated and randomly fragmented into 100 nucleotides or less, followed by the immunoprecipitation with 5 μg m^6^A antibody (ABclonal, China) or anti-Rabbit IgG which was linked to PierceTM Protein A/G Magnetic Beads (Thermo Fisher Scientific, USA). The bound RNAs were washed and eluted with elution buffer, purified through phenol-chloroform extraction. Further enrichment was calculated by qPCR and the corresponding m^6^A enrichment in each sample was calculated by normalizing to the input.

### RNA immunoprecipitation (RIP)

RIP analysis was performed using Magna RIP Kit (Millipore, Germany) according to manufacturer’s illustrations. Briefly, magnetic beads were mixed with 5 μg anti-METTL3 (Abclonal, China) or anti-Rabbit IgG before the addition of cell lysates (approximately 2 × 10^7^ cells for each sample). After washing with RIP wash buffer for three times, the beads were incubated with cell lysates at 4 °C overnight. The beads were washed with three times with wash buffer containing RNase inhibitor and Co-precipitated RNAs were eluted from immunoprecipitated complex and purified for further analysis using RT-qPCR. Relative enrichment was calculated as input%.

### Dual-luciferase reporter assay

Wild-type (WT) or mutant (Mut) TRPC3 were cloned into plasmid synthesized by Genechem (China) which was comprised of firefly luciferase and renilla luciferase. For mutant plasmid, Adenosine (A) in m^6^A motif was replaced by Thymine (T). The dual-luciferase assays were performed after transfection for 48 h by using Dual-Luciferase Reporter Assay kit (Yeasen, China) according to manufacturer’s protocol.

### Measurement of intracellular calcium ion concentration

Measurement of intracellular calcium release was performed as previously described [44]. Cells were seeded in a 4-well imaging chamber. After confluence to 70%, the cells were loaded with 5 μM Fluo-4 AM (Beyotime, China) Ca^2+^ indicator and incubated at 37 °C for 30 min. Thereafter, cells washed and incubated with HEPES buffer containing 1.8 mM Ca^2+^. The confocal microscope FV1000 (Olympus, Japan) was used to detect the fluorescence intensity every 10 s. Fluo-4 was excited at 488 nm, and data were expressed normalized to baseline fluorescence (*F*/*F*0).

### CCK-8 assay

Ovarian cancer cells were transfected with plasmid or siRNA for 24 h. Then an appropriate quantity of cells was seeded in 96-well plates. CCK-8 solution (Dojindo, Japan) was added into each well and incubated for 2 h at 0, 24, 48, 72, and 96 h after adhering to the plates. Optical density 450 nm value in each well was determined by a spectrophotometer reader (Thermo Fisher Scientific, USA).

### Statistical analysis

Statistical analyses were conducted with the SPSS 22.0 and GraphPad Prism 8.0 software. All experiments were performed in triplicate. Measured data were represented as the mean ± SD. One-way analysis of variance (ANOVA) or two-tail Student’s *t* test was applied to compare quantitative data, while the nonparametric *χ*2 test was used to analyze qualitative data. *P* values for each analysis are marked on figures, and the level of statistical significance was defined to *P* < 0.05 (*P < 0.05; **P < 0.01; ***P < 0.001; ****P < 0.0001).

## Supplementary information

supplementary materials

## Data Availability

All data created and analyzed during this current work are involved in this published article (and its supplementary information files).
