# Supplementary material for: PLAA suppresses ovarian cancer metastasis via METTL3-mediated m6A modification of TRPC3 mRNA
Source: Oncogene. 2022 Jul 22;41(35):4145–58. doi: 10.1038/s41388-022-02411-w (PMC9418004; doi:10.1038/s41388-022-02411-w)
Supplement: Supplementary file 1 — supplementary materials [file 41388_2022_2411_MOESM1_ESM.docx]

**PLAA suppresses ovarian cancer metastasis via METTL3-mediated m^6^A modification of TRPC3 mRNA**

Zhangjin Shen^1^, Lingkai Gu^1^, Yuwan Liu^1^, Lingfang Wang^1^, Jiawei Zhu^2^, Sangsang Tang^1^, Xinyi Wei^1^, Jiaying Wang^3^, Songfa Zhang^2^, Xinyu Wang^245^, Xiaodong Cheng^246^, Xing Xie^2^, Weiguo Lu^24^.

**Supplementary Figures**

**
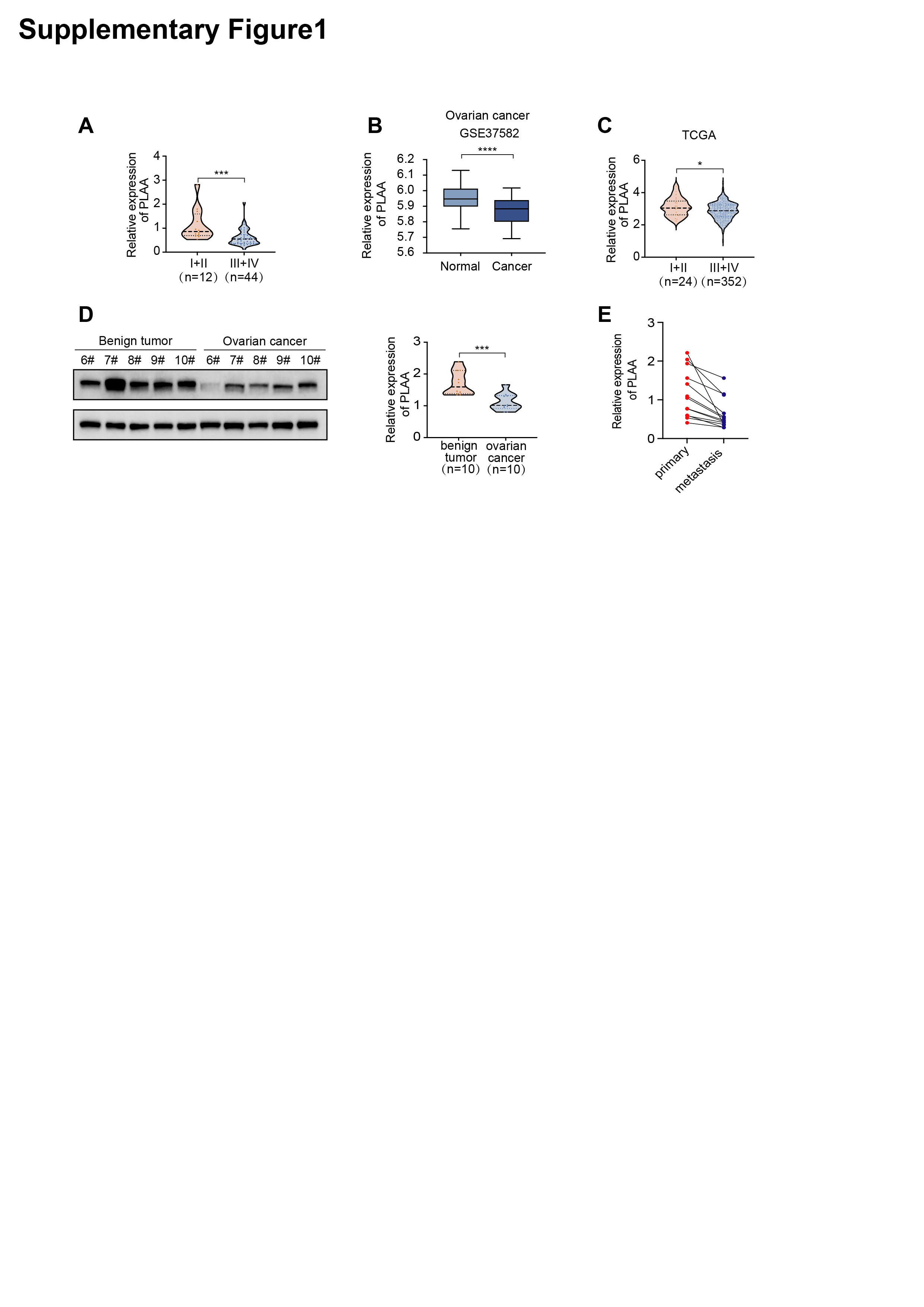
**

**Fig.S1 A** RT-qPCR analysis of PLAA expression in 56 tissue samples of ovarian cancer with different stages. **B** Relative expression of PLAA in ovarian cancer from GEO datasets. **C** Relative expression of PLAA in ovarian cancer tissues with different stages (data from TCGA). **D** Immunoblot analysis of PLAA expression in ovarian cancer tissues and ovarian benign tumor tissues. **E** RT-qPCR analysis of PLAA expression in ovarian cancer tissues and matched metastatic ovarian cancer tissues.

Data are representative of at least three independent experiments. *p < 0.05, **p < 0.01, ***p < 0.001，****p < 0.0001

**
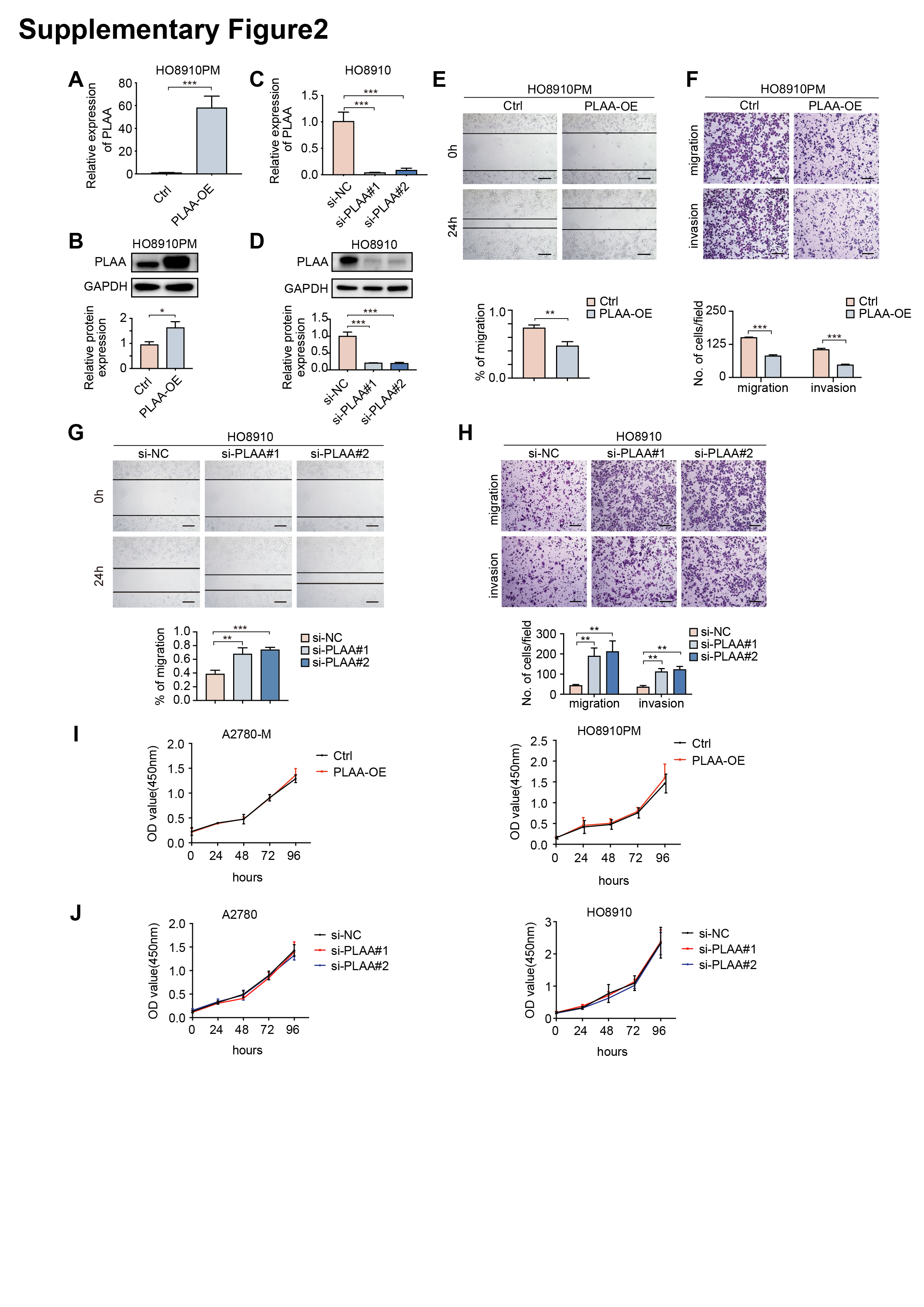
**

**Fig.S2 A-D** HO8910PM cells were transfected with PLAA overexpressing plasmid or empty plasmid **(A, B)**. HO8910 cells were transfected with two PLAA siRNAs or negative control **(C, D)**. PLAA expression was determined by RT-qPCR **(A, C)** or immunoblot analysis **(B, D)**. **E-F** HO8910PM cells were transfected with PLAA overexpressing plasmid or empty plasmid. Cellular migration and invasion were detected by wound healing **(E)** and transwell assay **(F)**. Scale bar, 100 μm. **G-H** HO8910 cells were transfected with two PLAA siRNAs or negative control. Cellular migration and invasion were detected by wound healing **(G)** and transwell assay **(H)**. Scale bar, 100 μm. **I-J** CCK-8 assays of PLAA-overexpressing **(I)** or -knockdown **(J)** cells were conducted to assess the proliferation ability.

Data are representative of at least three independent experiments. *p < 0.05, **p < 0.01, ***p < 0.001，****p < 0.0001

**
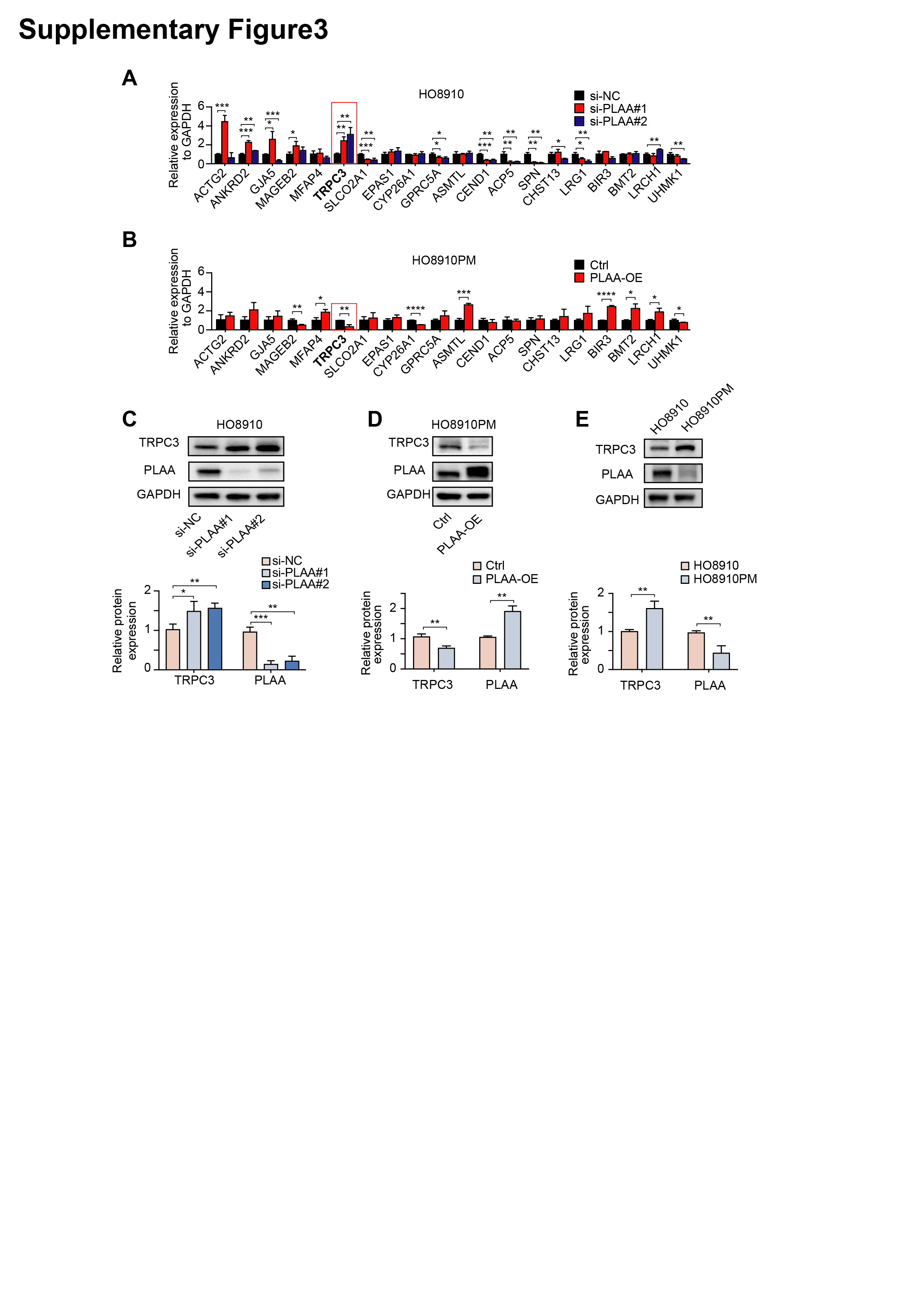
**

**Fig.S3 A-B** RT-qPCR analysis of differentially expressed mRNAs in HO8910 cells transfected with PLAA siRNAs **(A)** and HO8910PM cells transfected with PLAA plasmids **(B)**. The red frame shows TRPC3. **C** Immunoblot analysis of TRPC3 protein levels between HO8910 cells with PLAA knockdown and without. **D** Immunoblot analysis of TRPC3 protein levels between HO8910PM cells with PLAA overexpression and without. **E** Immunoblot analysis of PLAA and TRPC3 expression in HO8910PM and HO8910 cells.

Data are representative of at least three independent experiments. *p < 0.05, **p < 0.01, ***p < 0.001，****p < 0.0001

**
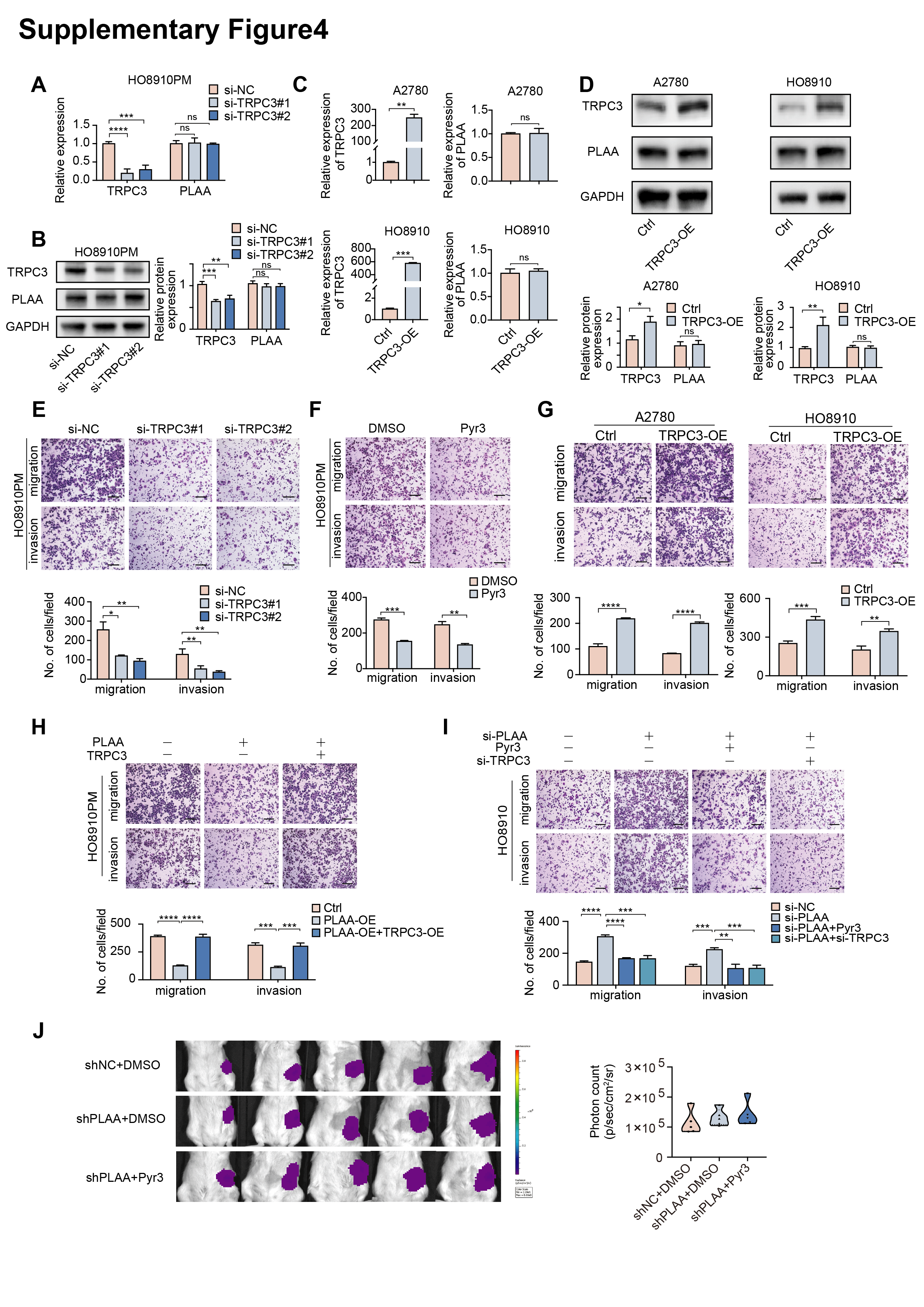
**

**Fig.S4 A-B** HO8910PM cells were transfected with two TRPC3 siRNAs or negative control. TRPC3 expression was determined by RT-qPCR **(A)** or immunoblot analysis **(B)**. **C-D** A2780 and HO8910 cells were transfected with TPRC3 overexpressing plasmid or empty plasmid. TRPC3 and PLAA expression was determined by RT-qPCR **(C)** or immunoblot analysis **(D)**. **E** HO8910PM cells were transfected with two TPRC3 siRNAs or negative control. Cellular migration and invasion were detected by transwell assay. Scale bar, 100 μm. **F** Cellular migration and invasion of HO8910PM cells treated with Pry3 or DMSO were detected by transwell assay. Scale bar, 100 μm. **G** A2780 and HO8910 cells were transfected with TPRC3 overexpressing plasmid or empty plasmid. Cellular migration and invasion were detected by transwell assay. Scale bar, 100 μm. **H-I** HO8910PM cells were transfected with PLAA plasmid, PLAA plasmid plus TRPC3 plasmid, and negative control, respectively **(H)**. HO8910 cells were transfected with PLAA siRNA, PLAA siRNA plus TRPC3 siRNA, PLAA siRNA plus Pyr3 treatment (2μM, 24h), and negative control, respectively **(I)**. Cellular migration and invasion were detected by transwell assay. Scale bar, 100 μm. **J** Bioluminescence images of SCID mice at day 7 before treatment with Pyr3 or DMSO. Photon count was shown on the right.

Data are representative of at least three independent experiments. *p < 0.05, **p < 0.01, ***p < 0.001，****p < 0.0001

**
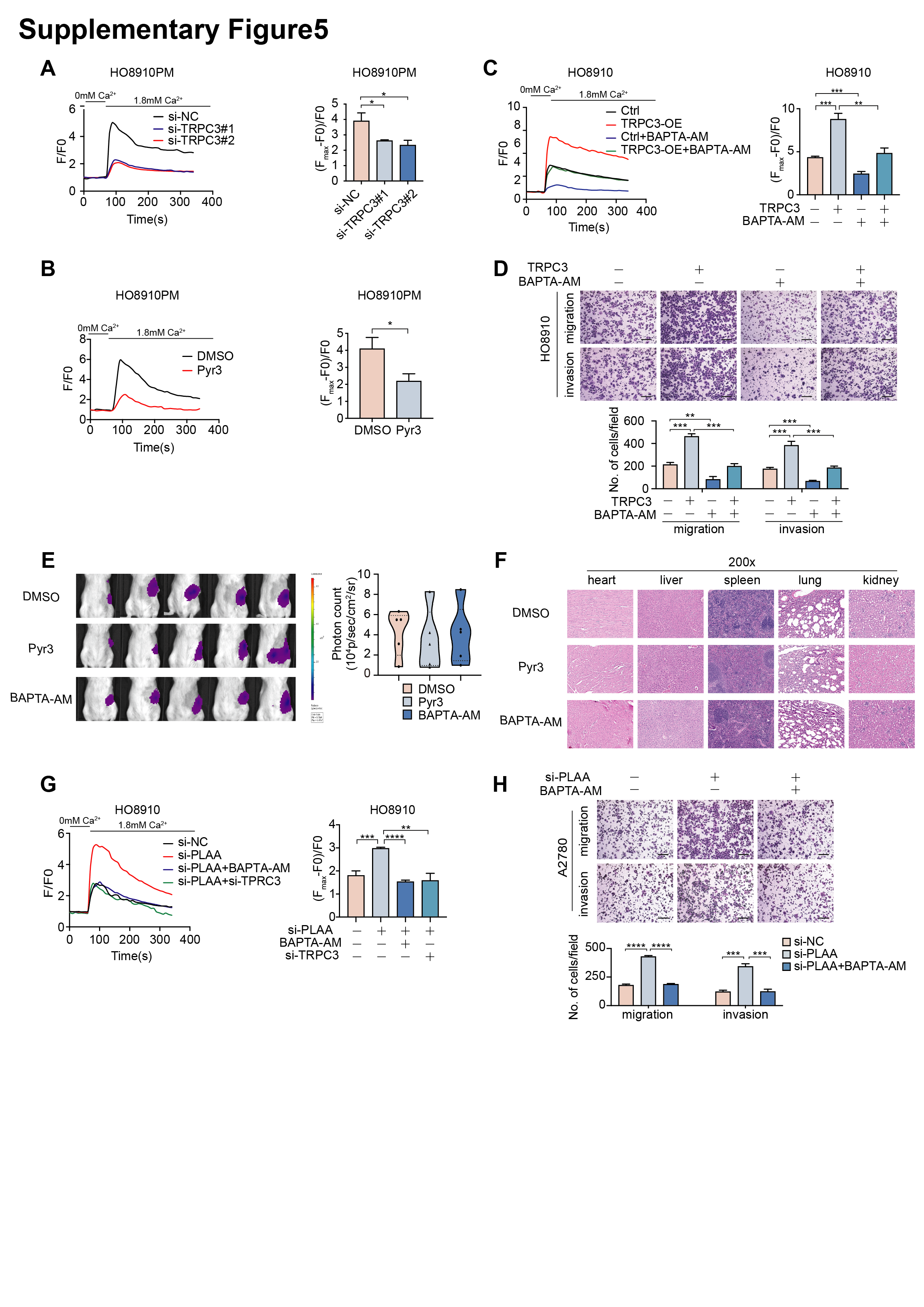

Fig.S5 A****-B** HO8910PM cells were transfected with two TRPC3 siRNAs **(A)** or treated with Pyr3 **(B)**. Addition of external calcium(1.8mM) led to an increase in fluorescence intensity and representative Ca^2+^ images tracing reflected changes of calcium concentration in the cytoplasm. F/F0: fluorescence normalized to baseline fluorescence. The net change in Ca^2+^ levels was normalized to (F_max_-F_0_)/F_0_. **C** HO8910 cells transfected with TRPC3 overexpressing plasmid or empty plasmid were treated with BAPTA-AM (20μg/mL, 24h) or not. Addition of external calcium(1.8mM) led to an increase in fluorescence intensity and representative Ca^2+^ images tracing reflected changes of calcium concentration in the cytoplasm. F/F0: fluorescence normalized to baseline fluorescence. The net change in Ca^2+^ levels was normalized to (F_max_-F_0_)/F_0_. **D** HO8910 cells transfected with TRPC3 overexpressing plasmid or empty plasmid were treated with BAPTA-AM (20μg/mL, 24h) or not. Cellular migration and invasion were detected by transwell assay. Scale bar, 100 μm. **E** Bioluminescence images of SCID mice at day 7 before treatment with Pyr3, BAPTA-AM or DMSO. Photon count was shown on the right. **F** Histological features of heart, liver, spleen, lung and kidney in the mouse model (200X magnifications). **G** HO8910 cells transfected with PLAA siRNA, PLAA siRNA plus BAPTA-AM (20μg/mL, 24h) treatment, PLAA siRNA plus TRPC3 siRNA, and negative control, respectively. Addition of external calcium(1.8mM) led to an increase in fluorescence intensity and representative Ca^2+^ images tracing reflected changes of calcium concentration in the cytoplasm. F/F0: fluorescence normalized to baseline fluorescence. The net change in Ca^2+^ levels was normalized to (F_max_-F_0_)/F_0_. **H** HO8910 cells transfected with PLAA siRNA, PLAA siRNA plus BAPTA-AM (20μg/mL, 24h) treatment, and negative control, respectively. Cellular migration and invasion were detected by transwell assay. Scale bar, 100 μm.

Data are representative of at least three independent experiments. *p < 0.05, **p < 0.01, ***p < 0.001，****p < 0.0001

**
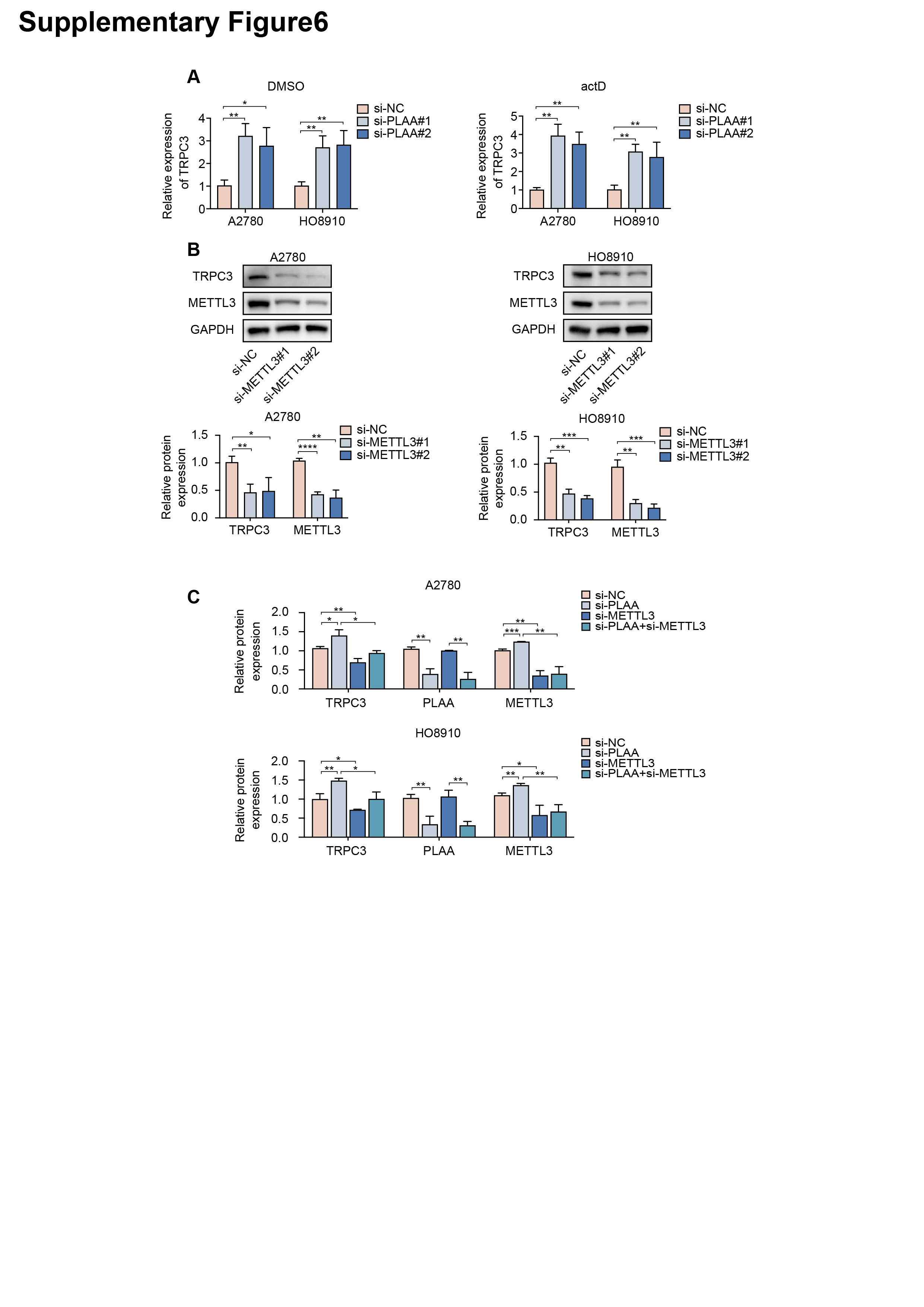
**

**Fig.S6 A** RT-qPCR analysis of TRPC3 mRNA expression in PLAA-knockdown A2780 and HO8910 cells with the treatment of actinomycin D (5 μg/ml) or not. **B** A2780 and HO8910 cells were transfected with METTL3 siRNA or negative control, respectively. TRPC3 and METTL3 expressions were determined by immunoblot analysis. **C** A2780 and HO8910 cells were transfected with PLAA siRNA, METTL3 siRNA, PLAA siRNA plus METTL3 siRNA, and negative control, respectively. Quantitation of the TRPC3, PLAA, and METTL3 protein expression.

Data are representative of at least three independent experiments. *p < 0.05, **p < 0.01, ***p < 0.001，****p < 0.0001

**Supplementary Tables**

**Table S1** Clinicopathologic characteristics of ovarian cancer associated with PLAA protein expression

| Case No. | Age | Gender | FIGO stage | CA125  (U/mL) | Ascites  (mL) | Lymph node metastasis | Time to recurrence(months) |
| --- | --- | --- | --- | --- | --- | --- | --- |
| 1 | 58 | female | IIB | 663.8 | 1500 | - | 57.5 |
| 2 | 36 | female | IC | 46.9 | 100 | - | 56.8 |
| 3 | 54 | female | IC | 73.7 | / | - | 56.8 |
| 4 | 49 | female | IIIA | 74.9 | 100 | - | 56.6 |
| 5 | 65 | female | IIIC | 409.4 | / | - | 55.7 |
| 6 | 46 | female | IIB | 28.1 | 200 | - | 31.0 |
| 7 | 53 | female | IIB | 35.3 | 200 | - | 54.0 |
| 8 | 52 | female | IIIC | 725.8 | 500 | - | 6.1 |
| 9 | 53 | female | IC | 164.2 | 80 | - | 53.7 |
| 10 | 43 | female | IIIC | 832.6 | 500 | - | 36.0 |
| 11 | 63 | female | IIIc | 247.3 | 100 | + | 52.7 |
| 12 | 69 | female | IC | 12.2 | 80 | - | 24.9 |
| 13 | 61 | female | IIIC | 1097 | 3000 | - | 4.1 |
| 14 | 49 | female | IIIC | 375 | 3000 | + | 37.0 |
| 15 | 49 | female | IIIC | 956.5 | 50 | - | 31.9 |
| 16 | 69 | female | IV | 745.9 | 1000 | - | 7.4 |
| 17 | 58 | female | IIIC | 2006 | 300 | + | 39.1 |
| 18 | 66 | female | IIIC | 108.5 | 30 | - | 30.0 |
| 19 | 57 | female | IIB | 593.2 | 100 | - | 48.7 |
| 20 | 55 | female | IIIC | 717.4 | 100 | - | 47.4 |
| 21 | 45 | female | IIA | 155.6 | 100 | - | 47.2 |
| 22 | 61 | female | IIIC | 62.2 | 50 | - | 46.9 |
| 23 | 54 | female | IIIC | 1872 | 800 | - | 29.0 |
| 24 | 63 | female | IIIC | 2830 | 30 | - | 36.0 |
| 25 | 45 | female | IIIC | 1827 | 1000 | + | 63.7 |
| 26 | 39 | female | IIIC | 3159 | 3500 | + | 35.0 |
| 27 | 50 | female | IIIC | 196.9 | 80 | + | 62.0 |
| 28 | 62 | female | IIB | 318.7 | 50 | - | 50.3 |
| 29 | 52 | female | IIIC | 150.3 | 200 | + | 40.1 |
| 30 | 51 | female | IIIA | 276.9 | 300 | - | 34.0 |
| 31 | 50 | female | IIIC | 812.3 | 200 | + | 18.8 |
| 32 | 61 | female | IIIC | 4374 | 500 | + | 45.2 |
| 33 | 34 | female | IC | 28.1 | 50 | - | 63.5 |
| 34 | 43 | female | IIIC | 3940 | 1000 | - | 36.0 |
| 35 | 55 | female | IIIC | 364.8 | 1000 | + | 33.0 |
| 36 | 57 | female | IIIB | 485 | 50 | - | 64.4 |
| 37 | 69 | female | IIC | 44.4 | 100 | - | 38.1 |
| 38 | 57 | female | IIIC | 1374 | 1500 | - | 50.3 |
| 39 | 47 | female | IIIC | 2217 | 5000 | - | 47.2 |
| 40 | 47 | female | IC | 2295 | 500 | - | 59.2 |
| 41 | 68 | female | IIIC | 363.1 | 100 | - | 24.4 |
| 42 | 47 | female | IIIC | 202.6 | 500 | - | 66.2 |
| 43 | 57 | female | IIA | 556.2 | 50 | - | 62.1 |
| 44 | 51 | female | IIIC | 2138 | 5000 | + | 9.7 |
| 45 | 52 | female | IV | 898.6 | 100 | - | 44.1 |
| 46 | 66 | female | IIB | 49.9 | 500 | - | 58.8 |
| 47 | 50 | female | IIA | 20 | 50 | - | 61.5 |
| 48 | 50 | female | IIIC | 123 | 4000 | + | 17.2 |
| 49 | 54 | female | IIB | 1054 | / | - | 40.1 |
| 50 | 77 | female | IIIC | 103.9 | 100 | - | 49.2 |
| 51 | 55 | female | IC | 286.3 | 300 | - | 64.2 |
| 52 | 41 | female | IIIC | 1154 | 100 | - | 68.4 |
| 53 | 50 | female | IIC | 103.4 | 50 | - | 17.8 |
| 54 | 39 | female | IV | 884.9 | 50 | - | 81.1 |
| 55 | 47 | female | IIIC | 9744 | 1000 | - | 80.3 |
| 56 | 56 | female | IIIC | 264.2 | 50 | - | 80.3 |
| 57 | 41 | female | IIC | 805.8 | 1500 | - | 54.3 |
| 58 | 64 | female | III | 1257 | 100 | - | 78.9 |
| 59 | 39 | female | IC | 41.8 | 50 | - | 56.3 |
| 60 | 58 | female | IC | 228.3 | 50 | - | 78.6 |
| 61 | 68 | female | IIA | 36.9 | 3500 | - | 78.1 |
| 62 | 54 | female | IIB | 740.6 | 50 | - | 45.2 |
| 63 | 56 | female | IIIC | 9868 | 100 | - | 55.4 |
| 64 | 57 | female | IIIC | 833.4 | 300 | - | 52.3 |
| 65 | 59 | female | IIIC | 2215 | 1500 | - | 76.3 |
| 66 | 67 | female | IA | 373.9 | 100 | - | 56.7 |
| 67 | 46 | female | IIIC | 929.5 | 500 | - | 52.3 |
| 68 | 46 | female | IC | 182.4 | / | - | 75.9 |
| 69 | 55 | female | IIIC | 1139 | 50 | - | 55.4 |
| 70 | 49 | female | IIA | 14 | 100 | - | 3.6 |
| 71 | 60 | female | IIA | 13 | 100 | - | 73.3 |
| 72 | 50 | female | IIIC | 770.3 | 3500 | - | 33.0 |
| 73 | 50 | female | IIIC | 508.7 | 3000 | + | 54.3 |
| 74 | 49 | female | IA | 258.1 | 50 | - | 46.2 |
| 75 | 57 | female | IIIA | 369.7 | 200 | - | 44.1 |
| 76 | 62 | female | IA | 67.5 | 100 | - | 71.1 |
| 77 | 64 | female | IIIC | 178.5 | 500 | - | 71.2 |
| 78 | 55 | female | IIC | 12 | 20 | - | 70.9 |
| 79 | 55 | female | IC | 18.9 | 100 | - | 70.7 |

**Table S2** Sequences of shRNAs and siRNAs against specific targets in this study.

| Item | Sequence | |
| --- | --- | --- |
| PLAA-shRNA-1 | Target Sequence | TGAAGGTGGACCATCATATAA |
| PLAA siRNA-1 | Sense (5’-3’) | GCGAGUGUCUUGAAGUAUATT |
|  | Antisense (5’-3’) | UAUACUUCAAGACACUCGCTT |
| PLAA siRNA-2 | Sense (5’-3’) | GCCAUAUAAUACCAGUGAUTT |
|  | Antisense (5’-3’) | AUCACUGGUAUUAUAUGGCTT |
| TRPC3 siRNA-1 | Sense (5’-3’) | GCCUUUAUGAUUGGCAUGUTT |
|  | Antisense (5’-3’) | ACAUGCCAAUCAUAAAGGCTT |
| TRPC3 siRNA-2 | Sense (5’-3’) | GGAUGACAGUGAUGUAGAATT |
|  | Antisense (5’-3’) | UUCUACAUCACUGUCAUCCTT |
| METTL3 siRNA-1 | Sense (5’-3’) | CGUCAGUAUCUUGGGCAAGTT |
|  | Antisense (5’-3’) | CUUGCCCAAGAUACUGACGTT |
| METTL3 siRNA-2 | Sense (5’-3’) | GGUUGGUGUCAAAGGAAAUTT |
|  | Antisense (5’-3’) | AUUUCCUUUGACACCAACCTT |
| Ctrl siRNA | Sense (5’-3’) | UUCUCCGAACGUGUCACGUTT |
|  | Antisense (5’-3’) | ACGUGACACGUUCGGAGAATT |

**Table S3** Sequences of primers used for qRT-PCR in this study.

| Item | Sequence | |
| --- | --- | --- |
| PLAA | Forward (5’-3’) | ACCCTCAAGTGACATCTACCC |
|  | Reverse (5’-3’) | GCCAGACTTTAGCAGTGGTGT |
| GAPDH | Forward (5’-3’) | GGAGCGAGATCCCTCCAAAAT |
|  | Reverse (5’-3’) | GGCTGTTGTCATACTTCTCATGG |
| TRPC3 | Forward (5’-3’) | AGAATGACTATCGGAAGCTCTCC |
|  | Reverse (5’-3’) | GGCAAGTTTGACACGACTTAATG |
| METTL3 | Forward (5’-3’) | TTGTCTCCAACCTTCCGTAGT |
|  | Reverse (5’-3’) | CCAGATCAGAGAGGTGGTGTAG |
| ACTG2 | Forward (5’-3’) | CATGTACGTCGCCATTCAAGC |
|  | Reverse (5’-3’) | TTGATGTCTCGCACAATTTCTCT |
| ANKRD2 | Forward (5’-3’) | CTGCGGAAGAAACGCAAGC |
|  | Reverse (5’-3’) | AGGGCCAGTGATCTCCTCG |
| GJA5 | Forward (5’-3’) | GCTGCCAGAATGTCTGCTAC |
|  | Reverse (5’-3’) | GGTACTCGTAAGAGCCAGAGC |
| MAGEB2 | Forward (5’-3’) | GCTGCGGGTGTTTCATCCA |
|  | Reverse (5’-3’) | TGGTTAGAGGATCTTCGCTTGG |
| MFAP4 | Forward (5’-3’) | TACCAGTCAGACGGCGTGTA |
|  | Reverse (5’-3’) | CCACTCGCAGCTCATACTTCT |
| SLCO2A1 | Forward (5’-3’) | TCGGTCTTCGGCAACATTAAG |
|  | Reverse (5’-3’) | GCTCTTGAAGTAGGCGCTGTA |
| EPAS1 | Forward (5’-3’) | TTGCTCTGAAAACGAGTCCGA |
|  | Reverse (5’-3’) | GGTCACCACGGCAATGAAAC |
| CYP26A1 | Forward (5’-3’) | ATGAAGCGCAGGAAATACGG |
|  | Reverse (5’-3’) | AGGAGTCGTGCAGGTTAGAGA |
| GPRC5A | Forward (5’-3’) | ATGGCTACAACAGTCCCTGAT |
|  | Reverse (5’-3’) | CCACCGTTTCTAGGACGATGC |
| ASMTL | Forward (5’-3’) | CTCTGGGAATACGTCCACAGC |
|  | Reverse (5’-3’) | GAAGTGGTTCAGCGGGAATCC |
| CEND1 | Forward (5’-3’) | CCAAGCCCTCGAAGAAGGAG |
|  | Reverse (5’-3’) | CTTCAGGTTGCTGTGGTTGT |
| ACP5 | Forward (5’-3’) | GACTGTGCAGATCCTGGGTG |
|  | Reverse (5’-3’) | GGTCAGAGAATACGTCCTCAAAG |
| SPN | Forward (5’-3’) | GCTGGTGGTAAGCCCAGAC |
|  | Reverse (5’-3’) | GGCTCGCTAGTAGAGACCAAA |
| CHST13 | Forward (5’-3’) | CCGGCATTTGGAAACAGAGC |
|  | Reverse (5’-3’) | CGGGTCCTGATCCAGGTCAT |
| LRG1 | Forward (5’-3’) | GGACACCCTGGTATTGAAAGAAA |
|  | Reverse (5’-3’) | TAGCCGTTCTAATTGCAGCGG |
| BIR3 | Forward (5’-3’) | AAGCTACCTCTCAGCCTACTTT |
|  | Reverse (5’-3’) | CCACTGTTTTCTGTACCCGGA |
| BMT2 | Forward (5’-3’) | GGCCGAAATACTGCTCGTG |
|  | Reverse (5’-3’) | TTCTCGGTACTTCTTGCGGAG |
| LRCH1 | Forward (5’-3’) | ACTCTGCACCCACTTCATCAT |
|  | Reverse (5’-3’) | GGTACGGGGAAATTCCTTCAAT |
| UHMK1 | Forward (5’-3’) | ACGCTGTCTGTTGCTTGAACT |
|  | Reverse (5’-3’) | GGCACAATGCTGTATCATCCAC |

**Table S4** Antibodies used in this study.

| Antigens | Manufacturer | Application |
| --- | --- | --- |
| Ubiquitin (AF1705) | Beyotime, China | 1:1000 for WB |
| METTL3 (A8370) | Abclonal, China | 1:1000 for WB, 5 µg per reaction for RIP |
| METTL14 (A8530) | Abclonal, China | 1:1000 for WB |
| WTAP (ab195380) | Abcam, USA | 1:1000 for WB |
| ALKBH5 (16837-1-AP) | Proteintech, USA | 1:1000 for WB |
| FTO (ab126605) | Abcam, USA | 1:1000 for WB |
| GAPDH (db106) | Diagbio, China | 1:2000 for WB |
| m^6^A (A17924) | Abclonal, China | 5 µg per reaction for Me-RIP |
| PLAA (ab133589) | Abcam, USA | 1:1000 for WB, 1:100 for IHC |
| TRPC3 (ACC-016) | Alomone，Israel | 1:200 for WB,  1:200 for IHC |
